# Unifying the roll waves

**DOI:** 10.1371/journal.pone.0310805

**Published:** 2024-11-19

**Authors:** Fabien Depoilly, Séverine Millet, Hamda Ben Hadid, Simon Dagois-Bohy, François Rousset

**Affiliations:** 1 LMFA, UMR5509, CNRS, Ecole Centrale de Lyon, INSA Lyon, Universite Claude Bernard Lyon 1, Villeurbanne, France; 2 INSA Lyon, CNRS, CETHIL, UMR5008, Villeurbanne, France; University of Science and Technology of China, CHINA

## Abstract

Free surface flows down a slope occur in various real-life scenarios, such as civil engineering, industry, and natural hazards. Unstable waves can develop at the free surface when inertia is sufficiently strong, indicated by the Reynolds number exceeding a critical value. Although this instability has been investigated for specific fluids with different rheologies, a common framework is still lacking to facilitate comparison among the various models. In this study, we investigate the linear stability of a generalized Newtonian fluid, where the viscosity $\eta (\dot{\gamma })$ remains unspecified. We meticulously construct new dimensionless quantities to minimize dependence on the rheology, and subsequently derive the Orr-Sommerfeld equation of stability for any generalized Newtonian fluid, which has never been done before. We conduct a long-wave expansion and generate a novel analytical expression for the wave celerity, along with the critical Reynolds number. The originality in this study is that the analytical expressions obtained are valid for any rheology, and are easy to compute from a rheological measurement or from a base flow profile measurement. These results are subsequently scrutinized using various shear-thinning, shear-thickening, and viscoplastic rheology models. They exhibit excellent agreement with experimental or numerical data as well as theoretical findings from existing literature. Furthermore, the novel analytical expressions enable a much more comprehensive investigation into the impact of rheology on stability. While our approach does not encompass singular or non-monotonous rheology, the analytical expressions derived from the long-wave expansion exhibit remarkable resilience and they continue to accurately predict both the wave speed and the instability threshold in such cases.

## Introduction

**Fig 1:**
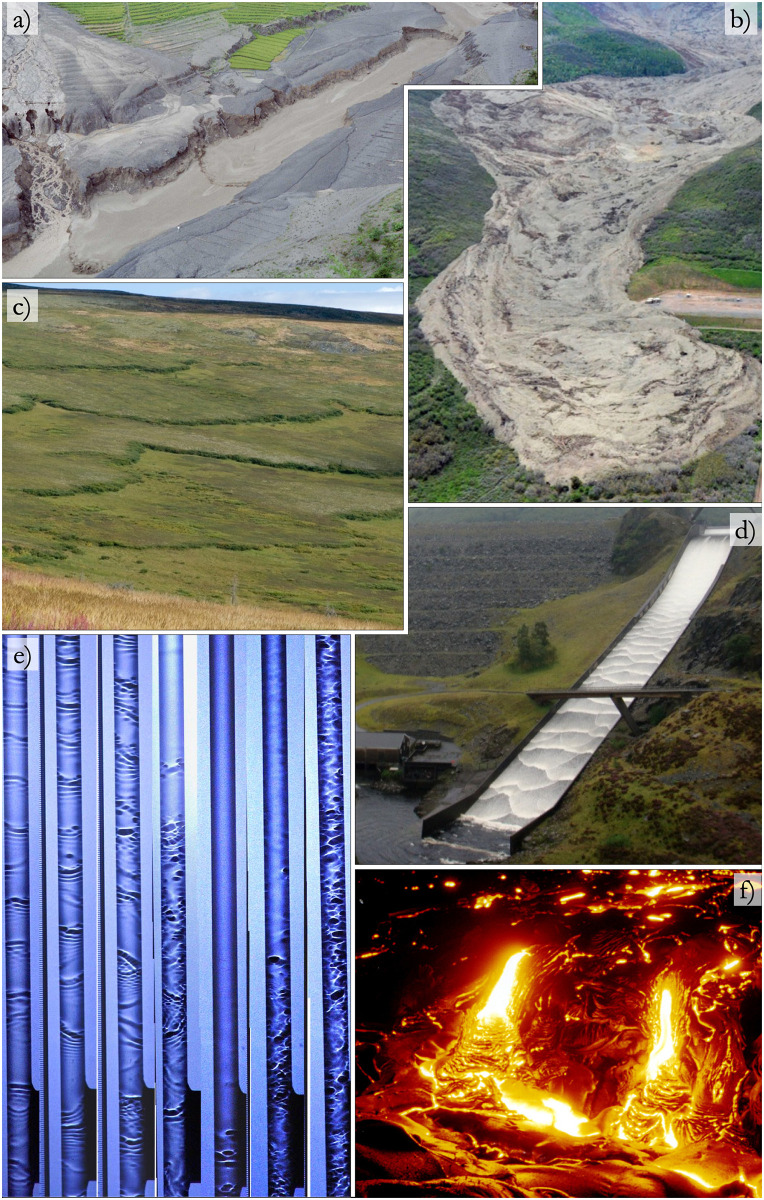
Examples of unstable surface flows down an inclined plane. a) Roll waves of a debris flow event in the Jang Jia Gully in China. (Photo: M. Arai, cropped) b) Giant mud flow in Colorado, 2014. (Photo: J. Coe, cropped) c) Solifluction terrace pattern near Eagle Summit, Alaska. (Photo D. Sikes, cropped) d) Roll waves in Llyn Brianne spillway, Wales. (Photo: J. Gibson, cropped) e) Roll waves in a vertical tube to augment mass transfer. (Photo: C.D. Park, cropped) f) Lava flow (2018) at Kilauea volcano, Hawai. (Photo: A. Glover, cropped). All the pictures above may have been modified for illustrative purposes.

Free surface flows driven by gravity down a slope manifest in various contexts, including civil engineering (such as spillways and aqueducts), industrial processes (like film coating, heat and mass transfer), and notably in geophysical phenomena (such as avalanches, landslides, lava flows, debris or mud flows). In these scenarios, the free surface may destabilize when the flow becomes sufficiently strong (see Fig 1), causing small perturbations to amplify into large roll waves. These waves may prove beneficial in certain processes like mass transfer [1, 2] but undesirable in others like surface coating [3] or natural hazards [4]. The destructive potential of large-scale roll waves underscores the importance of studying them, particularly in the context of risk assessment and structural integrity evaluation.

The evolution of these roll waves typically follows a well-established scenario: initial exponential growth during the linear regime, followed by amplitude saturation and stiffening of the wave front [5], and ultimately, secondary destabilization that may result in turbulence [6, 7]. Inertia is the main driving force behind this instability, with waves emerging only when the Reynolds number Re surpasses a critical threshold Re_*c*_ [8]. This critical value, which can be predicted through linear stability analysis, has been of particular interest in the literature.

At first, authors studied the stability of Newtonian fluids down a slope since the pioneering experiments of Kapitsa [9]; for that reason, roll waves are sometimes called Kapitsa waves. The critical Reynolds number, as well as the wave celerity, were established theoretically by studying the stability of a set of linear equations governing the perturbation fields [10, 11]. In the case where the perturbation fields are expressed as a stream function, the set of equations reduces to a single Orr-Sommerfeld equation [12], with free surface and no-slip boundary conditions. These predictions were verified experimentally using water-glycerin mixtures with Newtonian properties in a controlled experiment [13]. In parallel, other models considering flow quantities integrated over the layer thickness were developed, providing an evolution equation for the local flow height, but overestimating the critical Reynolds number when higher order terms were neglected [14–17].

In many real-life situations, the fluids involved deviate substantially from Newtonian rheology. Particularly in many geophysical flows, the fluids can exhibit shear-thinning behavior, with viscosity decreasing as the shear rate increases, or viscoplastic behavior, meaning they cannot flow when the stress is below an intrinsic material parameter, the yield stress [18]. This has prompted many authors to investigate the influence of rheology on roll waves, aiming to apply similar methods as those used in the Newtonian case.

The first non-Newtonian fluids considered were shear-thinning, with a power-law model describing their rheology [19, 20]. However, the analysis framework developed in the Newtonian case proved to be more challenging to apply than expected, primarily due to the diverging viscosity at zero shear rate. Various methods have been employed to address this issue, such as utilizing integrated quantities [21–25], introducing a small Newtonian layer near the free surface [26, 27], or employing a regularized rheology model, such as the Carreau law [28]. In the latter case, the critical Reynolds number and wave celerity were determined numerically and successfully compared to the results of other methods as well as experimental data [29].

The same modeling problems arose in the case of viscoplastic fluids, for which the viscosity divergence is aggravated by a stress discontinuity in the rheological model. The first successful modeling attempt was obtained by leaving out the part of the flow supposed to be undeformed, called pseudo-plug, considering it to be slowly sheared [30]. This model was later compared with experiments, and predicted successfully the critical Reynolds numbers and wave celerities, but failed at predicting the dispersion relations [31]. Other modeling methods were since attempted, such as using integrated quantities [32, 33], or regularized rheologies in a numerical model [34, 35], but so far only on Bingham fluids, a limiting subset of viscoplastic fluids. Integrated models were also notably used to analyze roll waves in the different but related context of granular chute flows [36].

Finally, in light of the recent interest in shear-thickening fluids, roll waves in these fluids have been investigated. Experimental and theoretical works have shown the coexistence of Kapitsa roll waves and a new type of instability, called Oobleck waves, due to the discontinuity in the rheology [37, 38]. These findings offer a promising new field of investigation, particularly in geophysics, where they could help understand unexplained features such as solifluction [39].

Roll waves in non-Newtonian fluids have also received recent attention in studies considering their interaction with complex geometries and physical effects, such as porous substrate, wavy bottom, thermal gradients or also surfactants [40–44]. These complex flows provide richer wave behaviors, and are a necessary step to describe real-life situations.

In this non-exhaustive, but representative state of the art, all the studies have in common that they present a linear stability analysis of long-wave unstable modes. This leads to a stability threshold, often a critical Reynolds number, driving the instability apparition. However, the change in rheology usually requires starting the analysis all over again, leading to results that are not easy to compare with each other, and with non-dimensional numbers that can differ dramatically from one study to another, even for the same rheology. A common mathematical framework is missing to unify these studies into a single formalism, which would make them easier to compare.

In fact, most of the fluids considered in these studies fall into the definition of generalized Newtonian fluids, that are fluids following a constitutive equation of the form:

$$\begin{array}{c} \tau =\eta \left(\dot{\gamma }\right)\dot{\gamma }, \end{array}$$ (1)

where *τ* is the shear stress, $\dot{\gamma }$ is the shear rate and *η* is the apparent viscosity.

In this paper, we propose a linear stability analysis of roll waves in a generalized Newtonian fluid, consistent with all the studies made before, and predicting the wave apparition threshold and celerity for any fluid of this category, including the ones that have never been studied yet.

The next section will present the main steps of the approach: after non-dimensional groups are defined, a perturbation equation is derived, leading to the full Orr-Sommerfeld equation with free-surface and no-slip boundaries. A long-wave expansion is performed, and analytical expressions for the wave celerity and the critical Reynolds number, valid for any generalized Newtonian fluid, are derived. Finally, these analytical expressions will be examined in the context of three families of non-Newtonian fluids: shear-thinning, shear-thickening, and viscoplastic. For each family, the predictions of this new model will be discussed and compared with the literature when possible.

## Stability analysis for a generalized newtonian fluid

In this section, we will present the main steps taken to study the linear stability of a generalized Newtonian fluid over an inclined plane. This section will focus on the main features of the model and leave some of the calculation details to the supporting information (S1 File).

### Rheological model

A generalized Newtonian fluid has its shear stress *τ* that depends only on its shear rate $\dot{\gamma }$, according to the constitutive relationship as

$$\begin{array}{c} \tau =F(\dot{\gamma })=\eta (\dot{\gamma })\dot{\gamma }, \end{array}$$ (2)

where *η* is the viscosity function of the fluid. Similarly one can express $\dot{\gamma }$ as a function of *τ* as

$$\begin{array}{c} \dot{\gamma }=G(\tau )=\Phi (\tau )\tau , \end{array}$$ (3)

where Φ is the fluidity function of the fluid [45]. Function *G* is the reciprocal function of *F*, and it only exists when *F* is strictly monotonous. By definition, viscosity and fluidity are inverse of each other, so that

$$\begin{array}{c} \eta (\Phi (\tau )\tau )\Phi (\tau )=1\;\;\text{and}\;\;\Phi (\eta (\dot{\gamma })\dot{\gamma })\eta (\dot{\gamma })=1. \end{array}$$ (4)

For example, the power-law fluid [19, 20] verifies the following relationships:

$$\begin{array}{c} \tau (\dot{\gamma })=\kappa \;{\dot{\gamma }}^{n}\;\;\;\text{and}\;\;\;\dot{\gamma }(\tau )={\left(\frac{\tau }{\kappa }\right)}^{\frac{1}{n}}, \end{array}$$ (5)

where *κ* is called the consistency and *n* the power-law index. The fluid is shear-thinning if *n* < 1, Newtonian if *n* = 1 and shear-thickening if *n* > 1. Its viscosity and fluidity are then

$$\begin{array}{c} \eta (\dot{\gamma })=\kappa \;{\dot{\gamma }}^{n-1}\;\;\;\text{and}\;\;\;\Phi (\tau )=\frac{\tau^{\frac{1}{n}-1}}{\kappa^{\frac{1}{n}}}. \end{array}$$ (6)

Finally, it should be highlighted that in the context of non-trivial flows, stress and strain rates are described by tensors of second order. The quantities $\dot{\gamma }$ and *τ* introduced so far should be seen as the second invariants of these tensors.

### Governing equations

**Fig 2:**
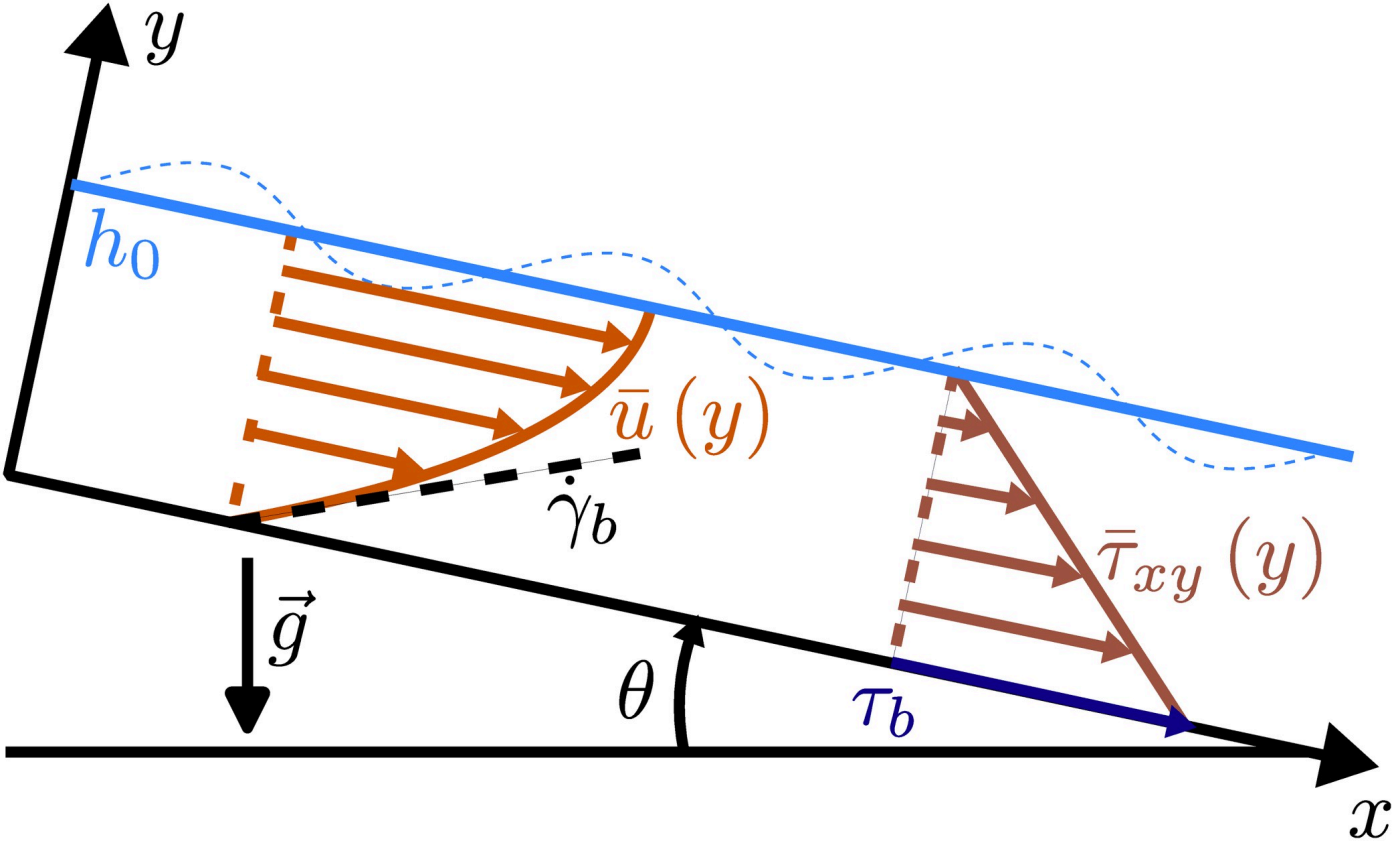
Scheme of the flow down an inclined plane, with base velocity and shear stress fields.

We consider the two-dimensional isothermal flow of an incompressible fluid driven by gravity down an infinite inclined plane of inclination *θ* with the horizon, as shown in Fig 2.

Let the origin of the Cartesian coordinate system (*x*, *y*) be placed at the bottom, with the *x*-axis oriented down the slope and the *y*-axis oriented normal to the wall toward the liquid side. The flow is governed by mass conservation and force balance:

$$\begin{array}{c} \nabla \cdot v=0, \end{array}$$ (7)

$$\begin{array}{c} \rho \frac{Dv}{Dt}=\nabla \cdot \sigma +\rho g, \end{array}$$ (8)

where ***v*** is the velocity field, having components (*u*, *v*) in the (*x*, *y*) system, *ρ* is the density, *D*/*Dt* is the material derivative and ***g*** is the acceleration due to gravity. For a generalized Newtonian fluid, the total stress tensor ***σ*** is given by:

$$\begin{array}{c} \sigma =-pI+\tau , \end{array}$$ (9)

where

$$\tau =\eta \left(\dot{\gamma }\right)\dot{\gamma },$$ (10)

and *p* is the pressure, ***τ*** is the deviatoric stress tensor, $\eta \left(\dot{\gamma }\right)$ is the viscosity, $\dot{\gamma }$ is the shear rate tensor and $\dot{\gamma }$ is its second invariant, called the shear rate, with

$$\dot{\gamma }=\left(\nabla v+\nabla v^{T}\right)\;\text{and}\;\dot{\gamma }=\sqrt{\frac{1}{2}\dot{\gamma }:\dot{\gamma }}.$$ (11)

As seen before, the rheology can be expressed in a reciprocal way as:

$$\dot{\gamma }=\Phi (\tau )\tau ,$$ (12)

with $\tau =\sqrt{\tau_{xx}^{2}+\tau_{xy}^{2}}$ the second invariant of the deviatoric stress tensor, called the shear stress.

To close this system of equations, conditions at the boundary are needed. We first suppose that there is no slip at the bottom so that ***v*** = **0** at *y* = 0, i.e.

$$\begin{array}{c} u=0\;\text{and}\;v=0\;\text{at}\;y=0. \end{array}$$ (13)

At the free surface *y* = *h*(*x*, *t*), the stress component tangent to the interface must vanish, and the normal stress has to balance surface tension effects, following Laplace’s law. These requirements are summarized by:

$$\begin{array}{c} \sigma \cdot n=(-p_{0}+2HS)n\;\;\text{at}\;\;\;y=h\left(x,t\right), \end{array}$$ (14)

where ***n*** is the outward unit normal, *H* is the mean curvature of the surface, *S* is the surface tension, and *p*_0_ is the atmospheric pressure.

There is one more boundary condition, called kinematic condition, relating the velocity field at the surface to the free surface position *h*(*x*, *t*):

$$\begin{array}{c} v=\frac{\partial h}{\partial t}+u\frac{\partial h}{\partial x}\;\text{at}\;y=h\left(x,t\right). \end{array}$$ (15)

### Base flow

We now solve Eqs 7–15 for the unperturbed flow, which is assumed steady and parallel to the wall. This will allow us to highlight some characteristic values that will later be useful for writing dimensionless equations. We use bars to denote the various quantities for the unperturbed flow. Assuming the layer thickness $\bar{h}(x,t)=h_{0}$ to be constant, the mass conservation and kinematic condition impose that the only non-zero velocity component $\bar{u}$ depends only on *y*. It follows from Eq 10 that only the off-diagonal components of the stress tensor are non-zero. Eq 8 simplifies greatly, and after considering the boundary conditions, we find the following affine relationships for pressure and stress:

$$\begin{array}{c} {\bar{\tau }}_{xy}=\rho g(h_{0}-y)\;\text{sin}\;(\theta ),\;\;\bar{p}=p_{0}+\rho g(h_{0}-y)\;\text{cos}\;(\theta ). \end{array}$$ (16)

Finally, to determine the velocity profile $\bar{u}(y)$, we have to resolve the equation given by the rheology:

$$\begin{array}{c} \frac{d\bar{u}}{dy}=\Phi ({\bar{\tau }}_{xy})\;{\bar{\tau }}_{xy}. \end{array}$$ (17)

To explicitly solve the base flow, one would need to specify the rheology of the fluid; however, the solution can be formally written in the general case as an integral over the thickness:

$$\begin{array}{c} \bar{u}(y)=\int_{0}^{y}(1-\frac{y_{1}}{h_{0}})\;\tau_{b}\;\;\Phi ((1-\frac{y_{1}}{h_{0}})\tau_{b})\;dy_{1}, \end{array}$$ (18)

where *τ*_*b*_ = *ρgh*_0_ sin (*θ*) is the shear stress at the bottom.

### Dimensionless equations

We now transform the equations by introducing dimensionless variables as well as dimensionless numbers that reflect the relative importance of the different terms. These dimensionless variables are built from the typical scales of the problem. At this stage, different choices can be made for the same variables, depending on which phenomenon one wants to emphasize. In this article, we want to compare the stability of fluids with different rheologies, so we choose typical scales related to their rheologies. The natural length scale is the height of the unperturbed flow *h*_0_, on which we build the dimensionless space variables. The unperturbed shear stress is maximum at the bottom, where it reaches the value *τ*_*b*_ = *ρgh*_0_ sin (*θ*), which we choose as the typical stress scale. The corresponding unperturbed shear rate at the bottom is given by the fluid rheology:

$$\begin{array}{c} {\dot{\gamma }}_{b}=G(\tau_{b})=\Phi (\tau_{b})\tau_{b}. \end{array}$$ (19)

We build a velocity scale by combining this shear rate with the unperturbed flow thickness as $u_{0}={\dot{\gamma }}_{b}h_{0}$. Note that this original velocity scale corresponds neither to the surface nor to the average fluid velocity, which are two other possible choices for the velocity scale found in the literature. Finally, we choose $\rho u_{0}^{2}$ to be the typical pressure scale, as it is usual in flows where inertia plays a role. To summarize, we introduce the following dimensionless variables:

$$\begin{array}{c} \hat{y}=\frac{y}{h_{0}},\;\;\hat{x}=\frac{x}{h_{0}},\;\;{\hat{\tau }}_{xx}=\frac{\tau_{xx}}{\tau_{b}},\;\;{\hat{\tau }}_{xy}=\frac{\tau_{xy}}{\tau_{b}},\;\;\hat{h}=\frac{h}{h_{0}},\\ \hat{u}=\frac{u}{{\dot{\gamma }}_{b}h_{0}},\;\;\hat{v}=\frac{v}{{\dot{\gamma }}_{b}h_{0}},\;\;\hat{t}=t{\dot{\gamma }}_{b},\;\;\hat{p}=\frac{p}{\rho {\dot{\gamma }}_{b}^{2}h_{0}^{2}}. \end{array}$$ (20)

It is then natural to define dimensionless shear stress and shear rate as

$$\begin{array}{c} \hat{\dot{\gamma }}=\sqrt{4{\left(\partial_{\hat{x}}\hat{u}\right)}^{2}+{\left(\partial_{\hat{y}}\hat{u}+\partial_{\hat{x}}\hat{v}\right)}^{2}},\;\;\hat{\tau }=\sqrt{{\hat{\tau }}_{xx}^{2}+{\hat{\tau }}_{xy}^{2}}, \end{array}$$ (21)

as well as dimensionless rheological relations, involving dimensionless viscosity and fluidity:

$$\begin{array}{c} \hat{\tau }=\hat{F}(\hat{\dot{\gamma }})=\hat{\eta }(\hat{\dot{\gamma }})\hat{\dot{\gamma }}\;\;\text{and}\;\;\hat{\dot{\gamma }}=\hat{G}(\hat{\tau })=\hat{\Phi }(\hat{\tau })\hat{\tau }, \end{array}$$ (22)

with

$$\begin{array}{c} \hat{\eta }(\hat{\dot{\gamma }})=\frac{\eta ({\dot{\gamma }}_{b}\hat{\dot{\gamma }})}{\eta ({\dot{\gamma }}_{b})}\;\;\text{and}\;\;\hat{\Phi }(\hat{\tau })=\frac{\Phi (\tau_{b}\hat{\tau })}{\Phi (\tau_{b})}. \end{array}$$ (23)

With this choice of non-dimensional variables, the base state calculated before takes a much simpler form:

$$\begin{array}{c} {\hat{\bar{\tau }}}_{xy}=1-\hat{y},\;\;\hat{\bar{p}}={\hat{p}}_{0}+\frac{\text{cot}(\theta )}{\text{Re}}(1-\hat{y}),\;\;\hat{\bar{h}}=1, \end{array}$$ (24)

where the Reynolds number is

$$\begin{array}{c} \text{Re}=\frac{\rho h_{0}u_{0}}{\eta ({\dot{\gamma }}_{b})}=\frac{\rho u_{0}^{2}}{\tau_{b}}. \end{array}$$ (25)

The base velocity field is simply expressed as

$$\begin{array}{c} \hat{\bar{u}}=\int_{0}^{\hat{y}}\hat{G}(1-{\hat{y}}_{1})d{\hat{y}}_{1}=\int_{0}^{\hat{y}}(1-{\hat{y}}_{1})\hat{\Phi }(1-{\hat{y}}_{1})d{\hat{y}}_{1}. \end{array}$$ (26)

Finally, the mass conservation and force balance equations write:

$$\begin{array}{c} \partial_{\hat{x}}\hat{u}+\partial_{\hat{y}}\hat{v}=0, \end{array}$$ (27)

and

$$\begin{array}{c} \text{Re}(\partial_{\hat{t}}\hat{u}+\hat{u}\partial_{\hat{x}}\hat{u}+\hat{v}\partial_{\hat{y}}\hat{u})=-\text{Re}\;\partial_{\hat{x}}\hat{p}+\partial_{\hat{x}}{\hat{\tau }}_{xx}+\partial_{\hat{y}}{\hat{\tau }}_{xy}+1, \end{array}$$ (28)

$$\begin{array}{c} \text{Re}(\partial_{\hat{t}}\hat{v}+\hat{u}\partial_{\hat{x}}\hat{v}+\hat{v}\partial_{\hat{y}}\hat{v})=-\text{Re}\;\partial_{\hat{y}}\hat{p}+\partial_{\hat{x}}{\hat{\tau }}_{xy}+\partial_{\hat{y}}{\hat{\tau }}_{yy}-\text{cot}\;(\theta ). \end{array}$$ (29)

### Perturbed flow

After obtaining the governing equations for dimensionless quantities, the goal is now to study the evolution of small perturbations around the base state. To do so, we will decompose the fields into a superposition of a base field and a small oscillating component, written as:

$$\begin{array}{c} \hat{u}=\hat{\bar{u}}+\tilde{u},\;\hat{v}=0+\tilde{v},\;\hat{p}=\hat{\bar{p}}+\tilde{p},\;\hat{\tau }=\hat{\bar{\tau }}+\tilde{\tau },\;\hat{h}=1+\tilde{h}, \end{array}$$ (30)

where a tilde denotes a dimensionless perturbed quantity. We then insert this decomposition into the governing equations and develop all the terms, neglecting quadratic terms in perturbed quantities. This linearization procedure is fairly standard in hydrodynamic stability problems and will not be detailed here. The originality lies in the perturbation of the rheological constitutive equation, which we write in reciprocal form to allow simpler expressions. In the end, the rheology of the fluid imposes the following relations between the perturbed fields:

$$\begin{array}{c} {\tilde{\tau }}_{xx}=2\delta \partial_{\hat{x}}\tilde{u}\;\text{and}\;{\tilde{\tau }}_{xy}=(\partial_{\hat{y}}\tilde{u}+\partial_{\hat{x}}\tilde{v})\gamma , \end{array}$$ (31)

with

$$\begin{array}{c} \delta (\hat{y})=\frac{1-\hat{y}}{{\hat{\bar{u}}}^{\prime }(\hat{y})}\;\text{and}\;\gamma (\hat{y})=\frac{-1}{{\hat{\bar{u}}}^{\prime \prime }(\hat{y})}. \end{array}$$ (32)

Here, the prime symbol denotes the derivative with respect to the vertical coordinate $\hat{y}$. To reduce the number of variables and equations, we introduce the stream function Ψ, which satisfies:

$$\begin{array}{c} \tilde{u}=\partial_{\hat{y}}\Psi ,\;\tilde{v}=-\partial_{\hat{x}}\Psi . \end{array}$$ (33)

We decompose the solution into normal modes of the form:

$$\begin{array}{c} \Psi =\psi (\hat{y})\;\text{exp}\;(i\alpha (\hat{x}-\hat{c}\hat{t}))\;\;\text{and}\;\;\tilde{h}=\xi \;\text{exp}\;(i\alpha (\hat{x}-\hat{c}\hat{t})), \end{array}$$ (34)

where *i* is the imaginary unit and *α*, $\hat{c}$, *ψ* and *ξ* are the dimensionless wave number, wave velocity, stream function amplitude and thickness amplitude of the perturbation. We study temporal stability, meaning *α* is assumed to be real and $\hat{c}$ is supposed to be complex. If its imaginary part is positive, the wave is amplified exponentially in time, i.e., unstable.

Substituting Eqs 33 and 34 into the set of equations, and eliminating the pressure $\hat{p}$, one obtains the following generalized Orr-Sommerfeld equation:

$$\begin{array}{c} i\alpha \text{Re}((\hat{\bar{u}}-\hat{c})(\psi^{\prime \prime }-\alpha^{2}\psi )-{\hat{\bar{u}}}^{\prime \prime }\psi )\\ ={\left(\gamma \psi^{\prime \prime }\right)}^{\prime \prime }+\alpha^{2}({\left(\gamma \psi \right)}^{\prime \prime }-4{\left(\delta \psi^{\prime }\right)}^{\prime }+\gamma \psi^{\prime \prime })+\alpha^{4}\gamma \psi . \end{array}$$ (35)

This equation governs the stability of the parallel flow of any fluid with a rheology given by a viscosity function $\hat{\eta }(\hat{\dot{\gamma }})$. It is determined by its parameters *δ* and *γ* defined in Eq 32, which depend only on the base flow, itself only dependent on the rheology (Eq 26). This is a new result and was never published before.

It reduces to the classical Orr-Sommerfeld equation for a Newtonian fluid, which can be obtained from Eq 35 by taking $\hat{\eta }=1$ and $\hat{\Phi }=1$ in the expressions. The boundary conditions associated with the free-surface problem under study are:

$$\begin{array}{c} \left\{ \begin{array}{c} \psi =0\\ \psi^{\prime }=0 \end{array}\;\;\;\text{at}\;\;\;\hat{y}=0,\right. \end{array}$$ (36)

and

$$\begin{array}{c} \left\{ \begin{array}{c} \gamma (\psi^{\prime \prime }+\alpha^{2}\psi )=\xi \\ i{\left(\alpha^{2}\gamma \psi +\gamma \psi^{\prime \prime }\right)}^{\prime }+\alpha \xi \;\text{cot}\;(\theta )+\alpha^{3}\xi T\;\text{Re}\\ \;\;\;\;\;+\alpha (\text{Re}(\hat{\bar{u}}-\hat{c})-4i\alpha \delta )\psi^{\prime }=0\\ \xi (\hat{c}-\hat{\bar{u}})=\psi \end{array}\;\text{at}\;\;\;\hat{y}=1,\right. \end{array}$$ (37)

with

$$\begin{array}{c} T=\frac{S}{\rho {\dot{\gamma }}_{b}^{2}h_{0}^{3}}. \end{array}$$ (38)

Several approaches could be taken to study this set of equations. In what follows, we will focus on the long-wave expansion, i.e., the limit when the wavelength is large compared to the thickness.

### Long-wave expansion

For the final step of this development, we will consider the long-wave limit *α* → 0. The stream function and the wave celerity can be expanded in power series of the small parameter *α* as:

$$\begin{array}{c} \psi =\psi_{0}+\alpha \psi_{1}+\alpha^{2}\psi_{2}+\cdots ,\;\hat{c}={\hat{c}}_{0}+\alpha {\hat{c}}_{1}+\alpha^{2}{\hat{c}}_{2}+\cdots , \end{array}$$ (39)

and the perturbation amplitude is normalised at $\hat{h}=1$. We focus here on the wave celerity, and after calculation (detailed in Supporting information), we find for the zeroth order in *α*:

$$\begin{array}{c} {\hat{c}}_{0}=1, \end{array}$$ (40)

and for the first order:

$$\begin{array}{c} {\hat{c}}_{1}=iA\left(\text{Re}-{\text{Re}}_{c}\right), \end{array}$$ (41)

where *A* is a positive pre-factor and Re_*c*_ is the critical Reynolds number. The latter reads:

$$\begin{array}{c} {\text{Re}}_{c}=\text{cot}\;(\theta )\frac{1-2\hat{q}}{1-4\hat{q}+2M+2K}, \end{array}$$ (42)

with $\hat{q}$ the dimensionless flow rate, M the average value of the square of the velocity (also referred to as form factor in certain contexts), and K the average value of the double integral of the squared shear rate:

$$\begin{array}{c} \hat{q}=\int_{0}^{1}\hat{\bar{u}}(y)dy,\;M=\int_{0}^{1}{\hat{\bar{u}}}^{2}(y)dy, \end{array}$$ (43)

$$\begin{array}{c} K=\int_{0}^{1}\int_{0}^{y}\int_{0}^{y_{1}}{\left({\hat{\bar{u}}}^{\prime }\left(y_{2}\right)\right)}^{2}dy_{2}\;dy_{1}\;dy. \end{array}$$ (44)

We emphasize that these expressions are new and have never been obtained before with this level of generality.

To conclude this section, we will briefly discuss these results and their implications. First, the expression found for the leading order of the wave celerity ${\hat{c}}_{0}$ is surprisingly simple with this choice of non-dimensional parameters. The velocity scale *u*_0_ chosen previously is, in fact, also the wave celerity at small wavenumber. If we revert to dimensional quantities, this means that the long-wave celerity corresponds to the kinematic wave celerity (see Supporting information):

$$\begin{array}{c} c_{0}=u_{0}=h_{0}{\dot{\gamma }}_{b}. \end{array}$$ (45)

As far as we know, no one has ever noticed that this result allows a new way to use the inclined plane setup as a rheometer. Indeed, the viscosity at the bottom can be calculated from the measured value of the wave celerity through

$$\begin{array}{c} \eta ({\dot{\gamma }}_{b})=\frac{\tau_{b}}{{\dot{\gamma }}_{b}}=\frac{\rho gh_{0}^{2}\;\text{sin}\;(\theta )}{c_{0}}. \end{array}$$ (46)

If *h*_0_ or *θ* varies, one could, in principle, explore the full stress-strain curve describing the rheology of the fluid, based on an independent measurement of the wave celerity. We want to emphasize that since measuring the wave celerity is generally non-invasive and reliable, this result could prove particularly useful in the context of in-situ observations, when the fluid is not directly accessible (industrial processes), or when a small sample may not be representative of the fluid rheology (debris or lava flows).

Secondly, the imaginary part of the wave celerity is found to be proportional to Re − Re_*c*_, and the flow is stable, neutrally stable, or unstable when the Reynolds number is respectively less, equal to, or greater than Re_*c*_. We note that Re_*c*_ is always proportional to cot(*θ*), and to free our discussion from this systematic *θ* dependency, we will use in the following sections the reduced Reynolds numbers defined as:

$$\begin{array}{c} {\text{Re}}^{\theta }=\text{Re}\;\text{tan}\;\left(\theta \right)\;\text{and}\;{\text{Re}}_{c}^{\theta }={\text{Re}}_{c}\;\text{tan}\;\left(\theta \right). \end{array}$$ (47)

The expressions of $\hat{q}$, M, and K involved in ${\text{Re}}_{c}^{\theta }$ can be expressed either with the base velocity field $\bar{u}(y)$ or with the rheological functions $\hat{\Phi }$ or $\hat{G}$, using Eq 26. As a consequence, one could establish Re_*c*_ with a measurement of the base flow velocity profile, without knowing the fluid rheology. Independently, one could also calculate Re_*c*_ for any flow of given slope angle *θ* and height *h*_0_, with a single rheology measurement.

As mentioned previously, in the literature of falling films, other definitions of the Reynolds number, based on different velocity scales, can be found. To compare the result of Eq 42 to previous results, a conversion needs to be done. In the majority of the cases, the Reynolds number is based on the mean velocity or the surface velocity, and the conversion is straightforward: ${\text{Re}}_{c}^{\theta ,\text{mean}}=\hat{q}\;{\text{Re}}_{c}^{\theta }$ or ${\text{Re}}_{c}^{\theta ,\text{surf}}=\bar{u}(1){\text{Re}}_{c}^{\theta }$.

Finally, we stress that the new expression found for Re_*c*_ is an analytical expression, and even though it is not always possible to derive an explicit mathematical expression for $\hat{\bar{u}}$, $\hat{\Phi }$ or $\hat{G}$, the integrals can be evaluated numerically.

## Application to specific rheologies

We will now test our model, particularly the critical Reynolds number expression and the long-wave velocity, with shear-thinning, shear-thickening, and viscoplastic rheologies. We will compare our results with previous models and experiments when possible, but for some of the fluids we will examine, the roll wave instability has never been studied before, and the predictions are new.

For each fluid, the method will be the same:

1. Write dimensional and non-dimensional rheology laws.
2. Calculate the base flow.
3. Calculate the critical Reynolds number, explicitly or numerically.

To ease the reading, these three steps will not be detailed for every fluid in the main text, but can all be found in the Supporting information.

### Shear-thinning fluids

Shear-thinning or pseudoplastic fluids exhibit viscosity that decreases when the shear rate increases. They find applications across various industries such as food, cosmetics, and pharmaceuticals, where this property is often achieved through the addition of polymers like xanthan gum and carboxymethyl cellulose (CMC). Additionally, they play a role in natural phenomena such as mud flows, lava flows, and debris flows. Shear-thinning behavior often emerges as the first non-Newtonian property when gradually altering the chemical composition from that of a pure Newtonian fluid. This is evident in kaolin suspensions, where at intermediate concentrations, the fluid demonstrates shear-thinning characteristics [21].

Numerous rheological models have been proposed in the literature to describe the behavior of shear-thinning fluids [46–49]. In this discussion, we will focus on four models that are representative and offer insights applicable to other similar models.

#### Power-law model

The simplest and perhaps the most common rheological model used to describe shear-thinning fluids is the power-law, also known as the Ostwald model [20], with *n* < 1 in Eq 6. This model is highly popular in engineering applications because it allows for the analytical solution of a wide range of flow problems [47].

1. In this model, the viscosity given by Eq 6 is directly proportional to the shear rate raised to an exponent *n* − 1. The non-dimensional version of this equation is simply $\hat{\eta }(\hat{\dot{\gamma }})={\hat{\dot{\gamma }}}^{n-1}$ and the fluidity is given by $\hat{\Phi }(\hat{\tau })={\hat{\tau }}^{-1+1/n}.$
2. Eq 26 provides the base velocity field as
  $$\begin{array}{c} \hat{\bar{u}}=\frac{n}{n+1}(1-{\left(1-\hat{y}\right)}^{\frac{1}{n}+1}). \end{array}$$ (48)
  The surface velocity is $\hat{\bar{u}}(1)=n/\left(n+1\right)$, indicating that the long-wave celerity is ${\hat{c}}_{0}=(1+1/n)\hat{\bar{u}}(1)$, in agreement with [50].
3. Finally, Eq 42 gives:
  $$\begin{array}{c} {\text{Re}}_{c}^{\theta }=1+\frac{3n}{2}. \end{array}$$ (49)

**Fig 3:**
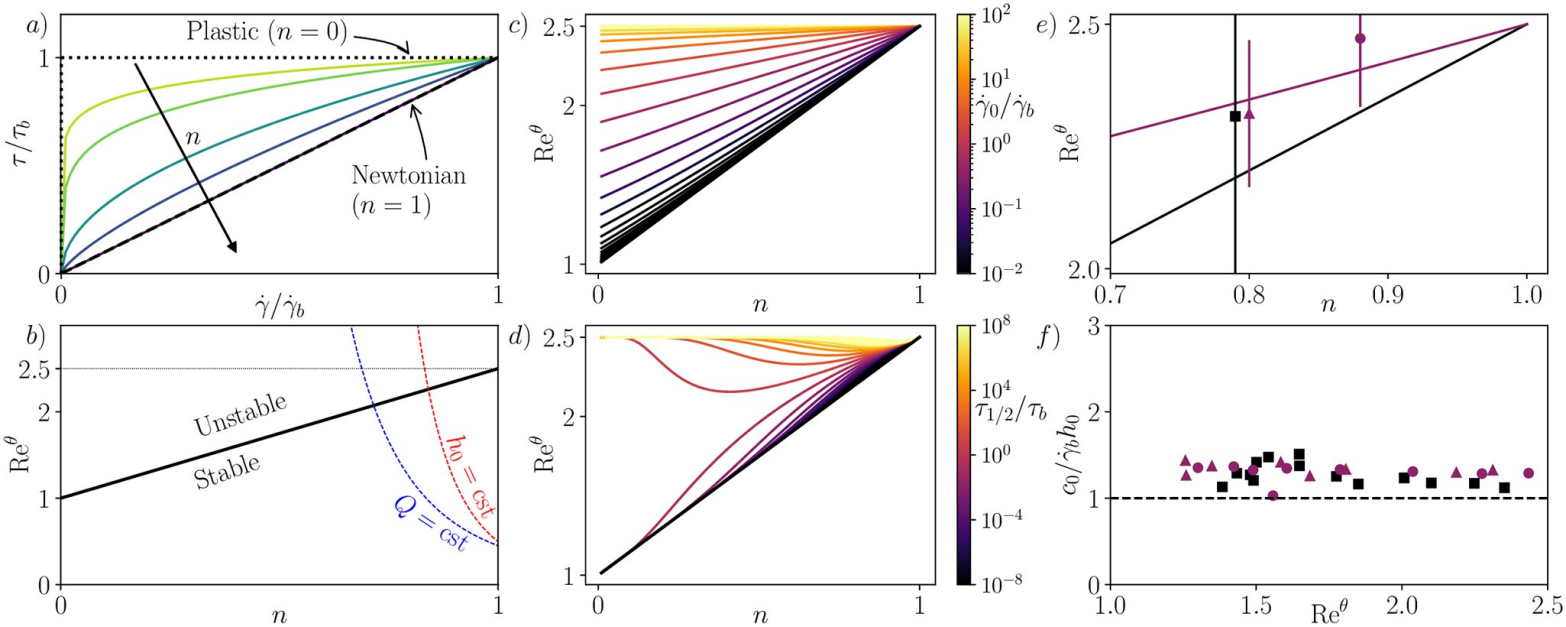
Stability results for shear-thinning fluids. a) Non-dimensional flow curves for various power-law fluids, with 0 < *n* < 1. b) Variation of the critical Reynolds number with *n* (black), and variation of the flow Reynolds number Re^*θ*^ with *n* when either *h*_0_ (red) or *Q* (blue) is kept constant. In both cases, when *n* decreases, Re^*θ*^ becomes greater than ${\text{Re}}_{c}^{\theta }$ and the flow is destabilized. c) Carreau fluid: ${\text{Re}}_{c}^{\theta }$ as a function of *n* for different regularization parameters ${\hat{\dot{\gamma }}}_{0}$. d) Ellis fluid: ${\text{Re}}_{c}^{\theta }$ as a function of *n* for different regularization parameters ${\hat{\tau }}_{1/2}$. e) Carreau fluid: ${\text{Re}}_{c}^{\theta }$ as a function of *n* for ${\hat{\dot{\gamma }}}_{0}=45$ (black) and ${\hat{\dot{\gamma }}}_{0}=1.6$ (violet). Points are experimental thresholds from [29], obtained for CMC and xanthan gum mixtures. f) Carreau fluid: wave celerity rescaled by $h_{0}{\dot{\gamma }}_{b}$, as a function of Re^*θ*^. Experimental data from [29].

This latter expression is consistent with the findings of [21, 30]. The two limits *n* → 1 and *n* → 0 correspond respectively to the Newtonian and the plastic behaviors (see Fig 3a). For the Newtonian fluid, we obtain ${\text{Re}}_{c}^{\theta }=5/2$, which aligns with the results of [8]. This corresponds to ${\text{Re}}_{c}^{\theta ,\text{mean}}=5/6$, a well-established result in the literature [10, 11]. The surface velocity is $\hat{\bar{u}}(1)=1/2$, indicating ${\hat{c}}_{0}=2\;\hat{\bar{u}}(1)$, another commonly encountered result in the literature on roll waves in Newtonian fluids. The plastic limit ${\text{Re}}_{c}^{\theta }=1$ is less straightforward to interpret. It corresponds to the limit of the base flow $\hat{\bar{u}}(\hat{y})\to 0$, which is attained for a pure plastic, where the thickness is too small for gravity to overcome the plastic limit. In this scenario, while the expression for ${\text{Re}}_{c}^{\theta }$ simplifies to ${\text{Re}}_{c}^{\theta }=1$, as long as the plastic does not yield, the actual Reynolds number of the flow remains 0, rendering the flow stable.

Fig 3b illustrates the decrease of ${\text{Re}}_{c}^{\theta }$ as *n* decreases. This has been previously interpreted as a destabilizing effect of the shear-thinning property [29, 30]. It’s worth noting that this reasoning holds when Re^*θ*^ is held constant, which may not be the most realistic condition. For comparison, Fig 3b also shows a typical variation of the Reynolds number as *n* varies, while keeping *h*_0_ or *Q*, the dimensional flow rate, constant. In both scenarios, the Reynolds number increases as *n* decreases, and the shear-thinning property has indeed a destabilizing effect. A more realistic model might need to account for how rheology changes, such as with a concentration parameter, and compare the trajectories obtained for Re^*θ*^ and ${\text{Re}}_{c}^{\theta }$, but this is beyond the scope of this paper.

In conclusion regarding this model, it’s worth noting that despite having a viscosity that diverges to infinity as $\dot{\gamma }\to 0$, we still obtain the well-established expression for the critical Reynolds number. However, while this is not problematic at orders 0 and 1 in *α*, the generalized Orr-Sommerfeld equation (Eq 35) for power-law fluids becomes inconsistent when considering terms of higher order in *α*. This is a known limitation of the power-law model, which can be unrealistic at low shear rates, particularly relevant for free surface flows. Various approaches exist to address this singularity, such as considering a regularized rheological model that approximates the power-law at high shear rates while maintaining a finite viscosity at zero shear rate.

#### Regularized power-law models

In the literature, various rheological models propose a regularized version of the power-law model. In this section, we will concentrate on two prominent ones: the Ellis model and the Carreau model.

1. For the Ellis model [51, 52], there is no direct expression of $\eta (\dot{\gamma })$, as the dimensional viscosity is expressed as a function of the (dimensional) shear stress:
  $$\begin{array}{c} \frac{\eta_{0}}{\eta }=1+{\left(\frac{\tau }{\tau_{1/2}}\right)}^{\frac{1}{n}-1}, \end{array}$$ (50)
  where *η*_0_ is the zero-shear-rate viscosity, *n* is a ‘power-law index’ and *τ*_1/2_ is the value of *τ* at which *η* = *η*_0_/2. The fluidity for such a fluid writes in a non-dimensional form:
  $$\begin{array}{c} \hat{\Phi }(\hat{\tau })=(1+{\left(\frac{\hat{\tau }}{{\hat{\tau }}_{1/2}}\right)}^{\frac{1}{n}-1})/(1+{\left(\frac{1}{{\hat{\tau }}_{1/2}}\right)}^{\frac{1}{n}-1}), \end{array}$$ (51)
  with ${\hat{\tau }}_{1/2}=\tau_{1/2}/\tau_{b}$. For the Carreau fluid, the dimensional viscosity is given by the expression:
  $$\begin{array}{c} \frac{\eta -\eta_{\infty }}{\eta_{0}-\eta_{\infty }}={\left(1+{\left(\frac{\dot{\gamma }}{{\dot{\gamma }}_{0}}\right)}^{2}\right)}^{\frac{n-1}{2}}, \end{array}$$ (52)
  with *η*_0_ the viscosity at zero shear rate, *η*_∞_ the viscosity at very high shear rate, *n* the rheological index and ${\dot{\gamma }}_{0}$ the shear rate over which the shear-thinning properties appear. To simplify, we will neglect the high shear rate viscosity (i.e., *η*_∞_ = 0). The non-dimensional expression of the viscosity becomes:
  $$\begin{array}{c} \hat{\eta }={\left(\frac{1+{\left(\hat{\dot{\gamma }}/{\hat{\dot{\gamma }}}_{0}\right)}^{2}}{1+{\left(1/{\hat{\dot{\gamma }}}_{0}\right)}^{2}}\right)}^{\frac{n-1}{2}}, \end{array}$$ (53)
  with ${\hat{\dot{\gamma }}}_{0}={\dot{\gamma }}_{0}/{\dot{\gamma }}_{b}$. In both models, a parameter describes the transition from Newtonian to shear-thinning behavior: ${\hat{\tau }}_{1/2}$ for the Ellis fluid, and ${\hat{\dot{\gamma }}}_{0}$ for the Carreau fluid. These parameters depend on the fluid characteristics (${\dot{\gamma }}_{0}$ and *τ*_1/2_), as well as on flow parameters (${\dot{\gamma }}_{b}$ or *τ*_*b*_).
2. For the Ellis fluid, it is possible to provide an explicit expression for the base flow $\bar{u}$ (see Supporting information), but for the Carreau fluid, it needs to be evaluated numerically.
3. Similarly, for the Ellis fluid, an explicit expression for ${\text{Re}}_{c}^{\theta }$ can be obtained (see Supporting information), but for the Carreau fluid, everything must be done numerically.

Fig 3c illustrates the variation of ${\text{Re}}_{c}^{\theta }$ with *n* for Carreau fluids at different ${\hat{\dot{\gamma }}}_{0}$. These results are obtained for the first time from the analytical general expression of Eq 42. When ${\hat{\dot{\gamma }}}_{0}$ is large, ${\text{Re}}_{c}^{\theta }$ is close to 5/2, which is the value for a Newtonian fluid. However, when ${\hat{\dot{\gamma }}}_{0}$ is small, ${\text{Re}}_{c}^{\theta }$ tends to 1 + 3*n*/2, representing the value for a power-law fluid. Despite the singularity arising at zero shear rate, the limit behavior of the rheological law at small ${\hat{\dot{\gamma }}}_{0}$ is reflected in the limit value of the critical Reynolds number. Our interpretation is that at small ${\hat{\dot{\gamma }}}_{0}$, there still exists a thin fluid layer at the surface where the rheology is close to Newtonian, thereby avoiding the singularity. However, this layer is too thin to significantly influence the stability threshold.

Similarly, Fig 3d illustrates the variation of ${\text{Re}}_{c}^{\theta }$ with *n* for Ellis fluids at different ${\hat{\tau }}_{1/2}$. Once again, the limit behaviors of the Newtonian and power-law fluids are observed at large and small values of ${\hat{\tau }}_{1/2}$, respectively, with the same interpretation as for the Carreau fluid. However, the main difference lies in the rate of convergence towards these limits: for the Carreau fluid, the convergence appears to be independent of *n* (uniform), whereas for the Ellis fluid, the convergence rate strongly depends on *n*. Other regularized models (such as Sisko, Cross, etc.) generally exhibit behavior similar to the Ellis model and present a non-uniform convergence rate towards the power-law or Newtonian limits. This convergence behavior can be utilized to distinguish between different regularized models, particularly in the context of extensive numerical simulations. In the absence of any other considerations, a model with uniform convergence should be preferred, such as the Carreau model in this case.

Finally, we compared our predictions for ${\text{Re}}_{c}^{\theta }$ and ${\hat{c}}_{0}$ with experimental data from [29] for different Carreau fluids, and we found a good agreement between them, as shown in Fig 3e and 3f.

To conclude this part, we should note that regularized models also enable the exploration of moderate waves. As we mentioned earlier, in the power-law model, the viscosity diverges, leading to inconsistencies in the generalized Orr-Sommerfeld equation when terms of order 2 or higher in *α* are considered. This limitation is not present in regularized models, allowing for the full resolution of Eqs 35–37 at any *α*. However, this aspect is not the focus of this article and will be explored in future work.

#### Eyring-Powell model

**Fig 4:**
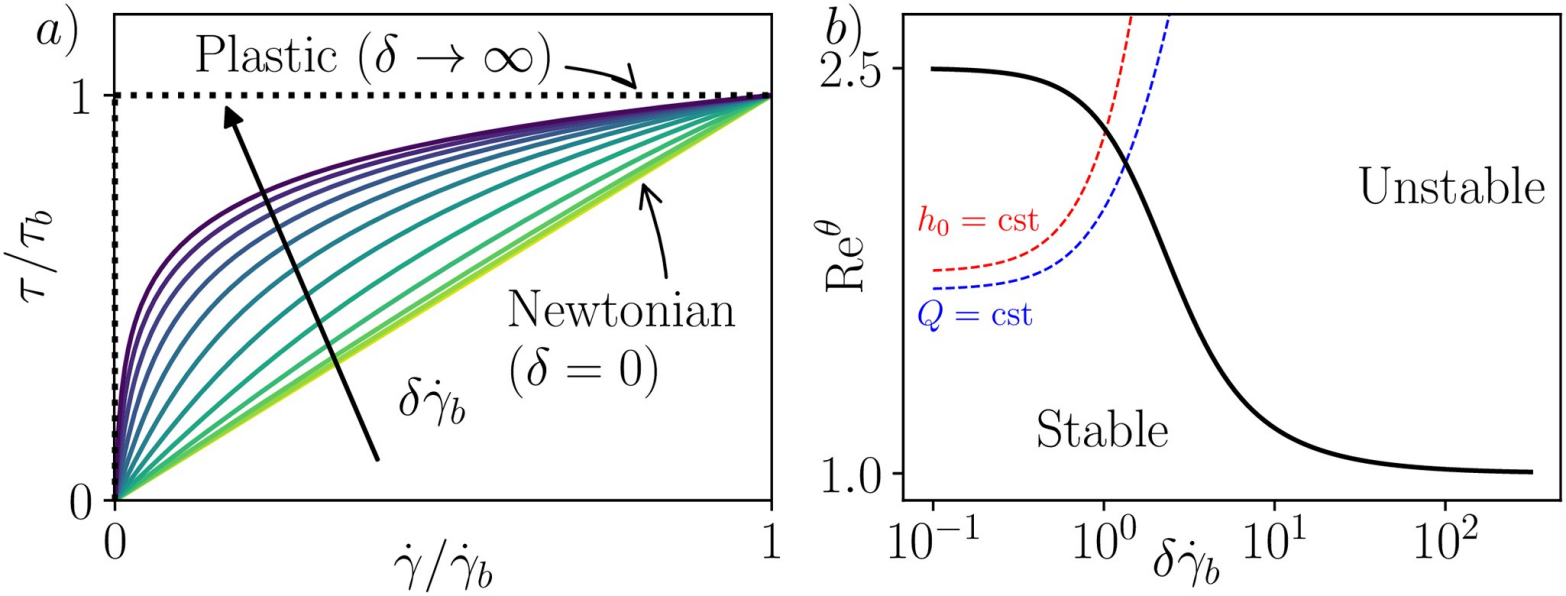
Stability results for Eyring-Powell fluids. a) Flow curves for Eyring-Powell fluids. At very high time scale *δ*, the fluid behaves like a pure plastic. b) Critical Reynolds number as function of the dimensionless time scale of the material (black). Dashed lines show the evolution of the Reynolds number with $\delta {\dot{\gamma }}_{b}$ when *h*_0_ = cst (red) and *Q* = cst (blue).

To conclude this section on shear-thinning fluids, we will now examine the Eyring-Powell fluid. This rheology was initially derived from a molecular theory [53, 54] and finds applications in modeling fluids confined at very small scales. While this rheological model may be less applicable to free surface flows, it remains noteworthy as one of the few models for shear-thinning fluids that does not stem from a regularization of the power-law. The viscosity expression for the Eyring-Powell fluid is as follows:

$$\begin{array}{c} \eta (\dot{\gamma })=\eta_{0}\;\frac{\text{arcsinh}(\delta \dot{\gamma })}{\delta \dot{\gamma }}, \end{array}$$ (54)

where *η*_0_ represents the zero-shear-rate viscosity and *δ* denotes a characteristic time of the material. The model predicts shear-thinning behavior for *δ* > 0, while both the Newtonian and plastic cases are retrieved as *δ* approaches 0 and + ∞, respectively (see Fig 4a).

In this context, it becomes feasible to provide an explicit expression for the non-dimensional fluidity $\hat{\Phi }\left(\hat{\tau }\right)$, alongside the base flow field $\hat{\bar{u}}$ and the critical Reynolds number ${\text{Re}}_{c}^{\theta }$, as functions of a dimensionless time constant $\delta {\dot{\gamma }}_{b}$ (see Supporting information).

Fig 4b illustrates the variation of ${\text{Re}}_{c}^{\theta }$ with $\delta {\dot{\gamma }}_{b}$ obtained from the general analytical expressions of Eqs 42 and 47. It is the first time that ${\text{Re}}_{c}^{\theta }$ is calculated with this specific rheology. Once more, we observe that a more shear-thinning fluid exhibits greater instability to gravity-driven flows, regardless of whether the Reynolds number Re^*θ*^, the flow height *h*_0_, or the flow rate *Q* are held constant. We observe the limiting cases of a Newtonian fluid and pure plastic behavior as *δ* tends towards 0 and + ∞, respectively, yielding ${\text{Re}}_{c}^{\theta }\to 5/2$ and ${\text{Re}}_{c}^{\theta }\to 1$, consistent with the viscosity law behavior in these limits. However, it is noteworthy that the convergence towards plastic behavior is logarithmic in $\delta {\dot{\gamma }}_{b}$ for the viscosity law, whereas it is faster for ${\text{Re}}_{c}^{\theta }$, occurring in $1/\delta {\dot{\gamma }}_{b}$. In other words, the plastic limit for free surface flows is reached more rapidly than in other configurations.

### Shear-thickening fluids

**Fig 5:**
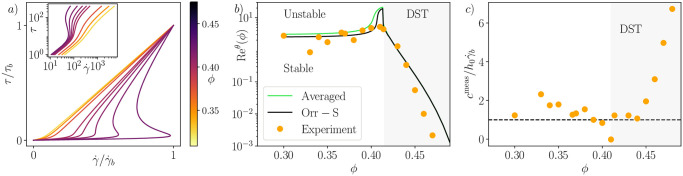
Stability results for shear-thickening fluids. a) Wyart-Cates rheology. b) ${\text{Re}}_{c}^{\theta }$ as a function of *ϕ* for shear-thickening suspensions. Black line: present model, green line and points: model and experiments from [38] for the Kapitsa mode only. Experimental data was measured at various slope angles, whereas both models were calculated at *θ* = 10°. c) Points: measured surface wave velocity from [38], normalised by $u_{0}={\dot{\gamma }}_{b}h_{0}$, dashed line: our model prediction.

Shear thickening fluids exhibit an increase in viscosity with the imposed shear rate (or stress). This behavior is predominantly observed in heterogeneous mixtures such as colloidal or non-colloidal suspensions, examples of which include cornstarch in water or silica in polyethylene-glycol solutions. Specifically, shear thickening occurs in dense particle suspensions where the particles interact via short-range repulsive forces of various origins (e.g., Brownian motion, electric repulsion, etc.). The contact between particles transitions from frictionless under low shear stress to frictional under high stress [55]. This transition induces a change in the jamming volume fraction, leading to sudden variations in viscosity. This behavior has been modeled for dense non-Brownian suspensions by Wyart and Cates [56] through a simple constitutive law:

$$\begin{array}{c} \eta =\frac{\eta_{s}}{{\left(\phi_{J}(\tau )-\phi \right)}^{2}}, \end{array}$$ (55)

where *η*_*s*_ is proportional to the solvent viscosity, *ϕ* is the particle volume fraction and *ϕ*_*J*_(*τ*) is the jamming volume fraction at which the viscosity diverges:

$$\begin{array}{c} \phi_{J}(\tau )=\phi_{0}(1-{\text{e}}^{-\tau^{*}/\tau })+\phi_{1}\;{\text{e}}^{-\tau^{*}/\tau }, \end{array}$$ (56)

where τ*, *ϕ*_0_ and *ϕ*_1_ are material constants (see Fig 5a). This constitutive law is known to successfully reproduce the different regimes observed for various *ϕ* [56], including continuous shear-thickening (CST), discontinuous shear-thickening (DST), and shear-jamming (SJ). In particular the the transition between CST and DST occurs at *ϕ* = *ϕ*_DST_. The investigation of roll waves in shear-thickening suspensions has been conducted in prior studies [37, 38], where an experimentally measured linear stability threshold has been identified. In the CST regime, roll waves may emerge when Re^*θ*^ > Re_*c*_^*θ*^. However, in the DST regime, surface waves arise from a distinct inertialess mechanism known as Oobleck waves. This mechanism is believed to be responsible for some solifluction patterns, which are anomalous wavy patterns observed in cold arctic soils [39].

In our model framework, the non-dimensional fluidity is expressed as:

$$\begin{array}{c} \hat{\Phi }(\hat{\tau })={\left(\frac{\phi_{J}(\hat{\tau })-\phi }{\phi_{J}(1)-\phi }\right)}^{2}, \end{array}$$ (57)

with

$$\begin{array}{c} \phi_{J}(\hat{\tau })=\phi_{0}(1-{\text{e}}^{{\hat{\tau }}^{*}\;/\hat{\tau }})+\phi_{1}\;{\text{e}}^{{\hat{\tau }}^{*}\;/\hat{\tau }}\;\;\text{and}\;\;{\hat{\tau }}^{*}=\tau^{*}/\tau_{b}. \end{array}$$ (58)

The critical Reynolds number cannot be explicitly calculated with this rheology; however, it is possible to evaluate it numerically from Eq 42. In experiments, the flow thickness *h*_0_ of a suspension at constant *ϕ* is gradually decreased until the flow becomes stable [38]. This process defines a critical Reynolds number ${\text{Re}}_{c}^{\text{exp}}\left(\phi \right)$, which we can predict through our approach by comparing Re and Re_*c*_ obtained at a given flow height *h*. The approach is similar to [38], but it is the first time it is done with the long-wave resolution of the full Orr-Sommerfeld equation.

Fig 5b compares this prediction with measurements from [38]. Despite potential experimental artifacts that could affect the comparison (such as finite channel thickness, finite frequency forcing, different angles, etc.), the prediction remains accurate for *ϕ* < *ϕ*_DST_. However, it overestimates ${\text{Re}}_{c}^{\theta }$ as *ϕ* approaches *ϕ*_DST_. Surprisingly, our prediction holds very well above *ϕ*_DST_, even though the instability is no longer driven by inertia. The reason lies in the appearance, when *ϕ* > *ϕ*_DST_, of a range of ${\hat{\tau }}^{*}$ for which ${\text{Re}}_{c}^{\theta }<0$. This implies that the growth rate $I\left(\alpha {\hat{c}}_{1}\right)=\alpha A\left(\text{Re}-{\text{Re}}_{c}\right)$ is always positive, even at Re = 0, indicating an inertialess instability. This instability arises when the basal shear stress exceeds a certain threshold depending on *ϕ*. It’s noteworthy that in the long wave regime, the inertialess growth rate is given by $-\alpha A{\text{Re}}_{c}=\alpha \;\text{cot}\;\left(\theta \right)\left(2\hat{q}-1\right)$, where $\hat{q}$ represents the dimensionless flow rate. The appearance of these Oobleck waves is contingent upon the condition $\hat{q}>1/2$, which requires a point of inflection in the base flow profile due to the S-shape of the rheology curve. However, the converse is not true, and hence we find that the volume fraction *ϕ* for which inertialess waves appear is slightly above *ϕ*_DST_ by a few tenths of a percent, a deviation likely beyond experimental accessibility. Furthermore, Fig 5c compares the measured wave speed to ${\dot{\gamma }}_{b}h_{0}$, and once again, our predicted scaling appears relevant for both inertial and Oobleck waves, particularly for volume fractions not too distant from *ϕ*_DST_. In conclusion, our results exhibit qualitative similarity to the model developed in [38], albeit with quantitative proximity to the experiments in the *ϕ* < *ϕ*_DST_ region, as expected from a rigorous resolution of the Orr-Sommerfeld equations compared to the Saint-Venant approximation. However, in the *ϕ* > *ϕ*_DST_ region, both models yield the same condition. Remarkably, in this region, our model adeptly captures the onset of instability, even though the flow curves are non-monotonous, which theoretically falls outside the scope of assumptions for a generalized Newtonian fluid.

### Viscoplastic fluids

In the last category under study, the fluids possess a yield stress *τ*_*y*_ at zero shear rate, and they resist flow when subjected to stresses below *τ*_*y*_. Strictly speaking, these fluids do not fall within the generalized Newtonian category, as the shear stress is not determined by the rheological law below the yield stress. However, we anticipate a different outcome compared to the previous section, as the relationship between strain rate and stress remains well-defined at all times.

#### Herschel-Bulkley models

The most common rheology used to describe the behavior of a viscoplastic fluid is the Herschel-Bulkley law, which defines the shear stress and viscosity as follows:

$$\begin{array}{c} \left\{ \begin{array}{cc} \dot{\gamma }=0 & \text{if}\;\tau <\tau_{y},\\ \tau =\tau_{y}+k{\dot{\gamma }}^{n} & \text{if}\;\tau ⩾\tau_{y}, \end{array}\;\;\right. \end{array}$$ (59)

so that

$$\begin{array}{c} \eta =\frac{\tau_{y}}{\dot{\gamma }}+k{\dot{\gamma }}^{n-1}\;\text{if}\;\tau ⩾\tau_{y}. \end{array}$$ (60)

**Fig 6:**
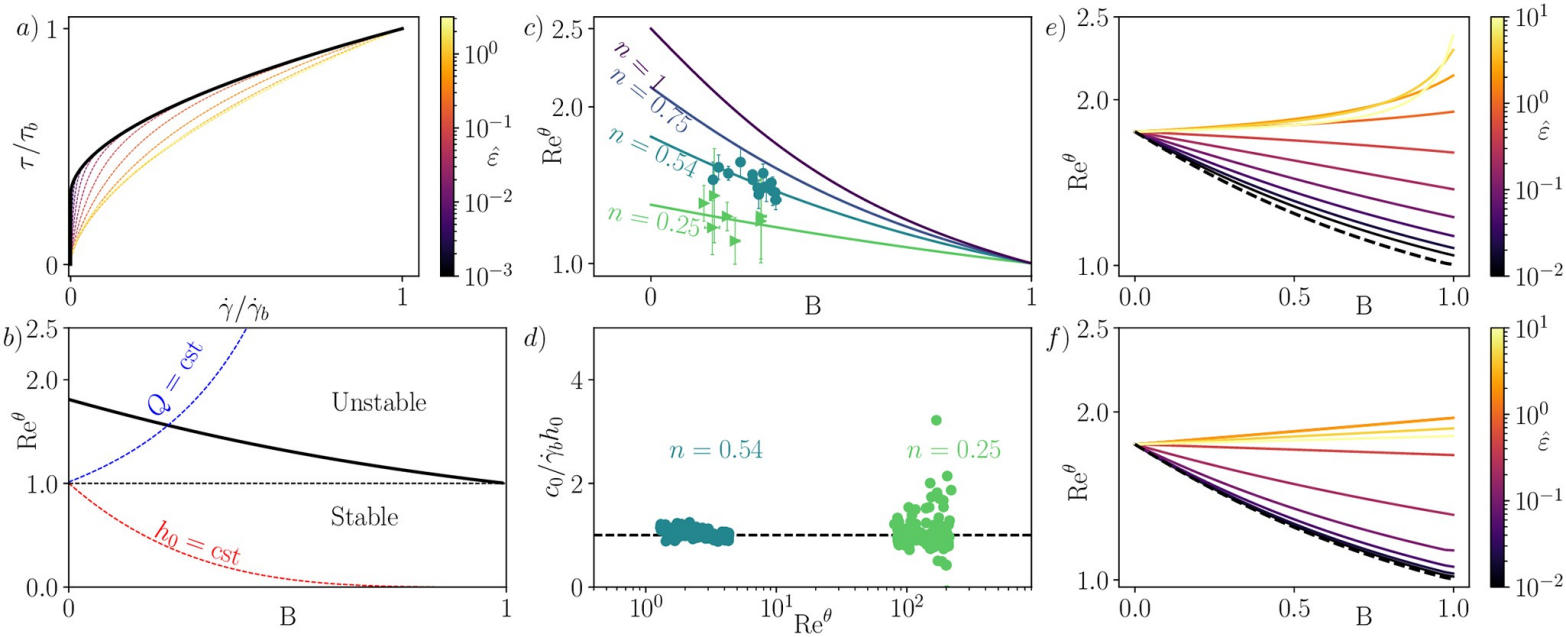
Stability results for viscoplastic fluids. a) Black line: non-dimensionalized flow curve for a Herschel-Bulkley fluid with *n* = 0.54 and B = 0.3. Colored dashed lines: flow curves for a regularized model (Papanastasiou), with the regularization parameter $\hat{\varepsilon }$ indicated in the color bar. b) Black line: ${\text{Re}}_{c}^{\theta }$ as a function of B for a Herschel-Bulkley fluid (*n* = 0.54). Dashed lines show the evolution of Re^*θ*^ with B when *h*_0_ = cst (red) and *Q* = cst (blue). In the former case, the yield stress has a stabilizing effect, but not in the latter. c) ${\text{Re}}_{c}^{\theta }$ as a function of B for Herschel-Bulkley fluids at different *n*. Points: experimental results in [31] obtained for Carbopol™ 980 (*n* = 0.54 in dark green) and kaolin suspensions (*n* = 0.25 in light green). Experimental data have been rescaled to match the Reynolds number definition in the present paper. d) Wave celerity measured in [31] for Carbopol™ 980 (*n* = 0.54 in dark green) and kaolin suspensions (*n* = 0.25 in light green), rescaled by $h_{0}{\dot{\gamma }}_{b}$. Each set of points corresponds to a single point in panel c). e), f) ${\text{Re}}_{c}^{\theta }$ as a function of B for regularized viscoplastic models of Williamson (e) and Papanastasiou (f). The corresponding regularization parameter $\hat{\varepsilon }$ is indicated in the color bar.

The bottom shear rate is then:

$$\begin{array}{c} {\dot{\gamma }}_{b}={\left(\frac{\tau_{b}-\tau_{y}}{k}\right)}^{\frac{1}{n}}={\left(\frac{\tau_{b}\left(1-B\right)}{k}\right)}^{\frac{1}{n}}, \end{array}$$ (61)

with B = *τ*_*y*_/*τ*_*b*_ the Bingham number, which compares the yield stress to the bottom shear stress. In non-dimensional terms, the rheology can be expressed as follows:

$$\begin{array}{c} \left\{ \begin{array}{cc} \hat{\tau }=B+\left(1-B\right){\hat{\dot{\gamma }}}^{n} & \;\text{if}\;\hat{\tau }⩾B,\\ \hat{\dot{\gamma }}=0 & \;\text{if}\;\hat{\tau }<B, \end{array}\right. \end{array}$$ (62)

as plotted in Fig 6a. When B = 1, the dimensional shear stress remains below *τ*_*y*_ everywhere, preventing any flow, akin to the plastic limit. When B = 0, we revert to the power-law constitutive relation. For *n* = 1, the fluid exhibits Bingham behavior, and B = 0, *n* = 1 corresponds to a Newtonian fluid. It is feasible to derive an explicit expression for the base flow and the critical Reynolds number, as detailed in the SI. The expression aligns with literature [30], albeit without the diverging factor present in (1 − B)^−2/*n*^, given the utilization of a distinct set of non-dimensional quantities.

Fig 6c depicts ${\text{Re}}_{c}^{\theta }$ as a function of B obtained from Eq 42 for various values of *n*. As B → 1 or *n* → 0, ${\text{Re}}_{c}^{\theta }\to 1$ for all *n* or all B, respectively, consistent with the expected plastic limit behavior. When B = 0, one has ${\text{Re}}_{c}^{\theta }=1+3n/2$ for the power-law fluid, with the special Newtonian case yielding ${\text{Re}}_{c}^{\theta }=5/2$ for *n* = 1. Furthermore, Fig 6c also displays stability thresholds measured in experiments [31], with Reynolds numbers recalculated to match the definition used in this article. We observe good agreement between Eq 42 and experimental data for two different fluids with distinct rheological indices. Moreover, in Fig 6d, we observe that the wave celerity measurements from the experiments in [31] also conform to the scaling proposed in Eq 45, once again for two different fluids with differing *n*. Additionally, our result elucidates the experimental correlation ${\text{Re}}_{c}^{\theta }\approx \left(1+3n/4\right)$ found in [31]: this simply arises as the average between the two limits obtained at B = 0 and B = 1.

As shown in Fig 6b, the critical Reynolds number systematically decreases as B increases, a phenomenon previously interpreted as the destabilizing effect of the yield stress on the flow [34]. Again, this interpretation is based on reasoning at constant Reynolds number. For comparison, we calculated Re^*θ*^ and examined how it varies when the yield stress *τ*_*y*_ changes, while either *h*_0_ or *Q* are kept constant. We represented these trajectories as a function of the dimensionless yield stress B in Fig 6b. When *h*_0_ = cst (equivalent to keeping the basal shear stress *τ*_*b*_ constant), the Reynolds number of the flow decreases much faster than the critical Reynolds number. In this scenario, the yield stress exhibits a clear stabilizing effect on the flow, consistent with claims made by other authors [30], albeit with a different non-dimensional scaling. On the other hand, when *Q* = cst, the Reynolds number grows and diverges as B → 1. In this case, the yield stress destabilizes the flow; however, this scenario may not be very realistic as it implies a diverging flow thickness.

To conclude, we assert that these results are consistent with the literature [30], albeit expressed using a different set of non-dimensional quantities, leading to a distinct apparent scaling of ${\text{Re}}_{c}^{\theta }$ with B. It is noteworthy that no assumptions were made regarding the film thickness or angle in our analysis, and there was no requirement for the pseudo-plug model as done previously [30]. However, it’s essential to acknowledge that outside of the long-wave approximation, our set of equations becomes inconsistent due to viscosity divergence, rendering us unable to solve the generalized Orr-Sommerfeld equation 35. In Herschel-Bulkley fluids, issues related to viscosity divergence are not limited to this specific flow and pose significant challenges in modeling flows of viscoplastic fluids. Similar to shear-thinning fluids, several attempts have been made to regularize the Herschel-Bulkley rheological law. In the next subsection, we will explore the impact of these regularizations on the stability threshold.

#### Regularized models: Williamson, Papanastasiou

We conclude this section on viscoplastic fluids by examining two regularized viscoplastic rheologies: the Williamson model [57] and the Papanastasiou model [58]. The Williamson model involves introducing a cutoff *ε* in the diverging viscosity term associated with the yield stress. The viscosity is then defined as follows:

$$\begin{array}{c} \eta (\dot{\gamma })=k{\dot{\gamma }}^{n-1}+\frac{\tau_{y}}{\varepsilon +\dot{\gamma }}, \end{array}$$ (63)

where *k*, *τ*_*y*_ and *n* are the same as in the Herschel-Bulkley law. In the Papanastasiou model, regularization is achieved by introducing an exponential term in front of the yield stress term. This results in the following expression for the viscosity:

$$\begin{array}{c} \eta (\dot{\gamma })=k{\dot{\gamma }}^{n-1}+\frac{\tau_{y}(1-{\text{e}}^{-\dot{\gamma }/\varepsilon })}{\dot{\gamma }}. \end{array}$$ (64)

In both models, the viscosity approaches the Herschel-Bulkley viscosity at high $\dot{\gamma }$, while at low $\dot{\gamma }$, the diverging term associated with the yield stress is bounded. The transition between these two behaviors occurs when $\dot{\gamma }\approx \varepsilon$, i.e., when $\hat{\dot{\gamma }}\approx \hat{\varepsilon }$ in non-dimensional form where $\hat{\varepsilon }=\varepsilon /{\dot{\gamma }}_{b}$. Originally, these models were proposed only for *n* = 1 as an approximation of a Bingham fluid, and were later extended to model a Herschel-Bulkley fluid with *n* ≠ 1. However, it becomes apparent that these propositions are somewhat simplistic, as the viscosity still diverges due to the power-law term. A potential solution could involve regularizing the power-law term as well, for instance, using a Carreau model. However, this aspect will be addressed in future research.

Fig 6e and 6f illustrate the critical Reynolds number ${\text{Re}}_{c}^{\theta }$ obtained for the first time with these regularized laws as a function of B, for a fixed intermediate value of *n* = 0.54 corresponding to the Carbopol fluid used in [31], and for different values of the regularization parameter $\hat{\varepsilon }$. In both models, the critical Reynolds number ${\text{Re}}_{c}^{\theta }$ approaches the ${\text{Re}}_{c}^{\theta }$ calculated for a Herschel-Bulkley fluid as the regularization parameter becomes sufficiently small. In a sense, the Herschel-Bulkley fluid, while not strictly a generalized Newtonian fluid, can be viewed as the uniform limit of a series of regularized rheologies that all satisfy the appropriate set of hypotheses. This result contrasts with the pseudo-plug theory [30], where the base flow is regularized instead of the rheology. Additionally, we observe that the Papanastasiou regularization converges more rapidly than the Williamson regularization, likely due to the presence of the exponential term. However, apart from this, no clear advantage is evident for preferring one regularization over the other, possibly because they lack a clear physical micro-mechanism.

## Conclusions

In this article, we introduced a unified model to describe the roll waves instability for fluids of various rheologies within the family of generalized Newtonian fluids. For the first time, we derived the Orr-Sommerfeld equation for a generalized Newtonian fluid, elucidating the stability of a parallel flow in 2D. By combining this equation with the appropriate boundary conditions and employing a long-wave expansion, we derived two analytical expressions: one for the wave speed and another for the critical Reynolds number, which quantifies the onset of this instability.

We subsequently validated our model with fluids exhibiting various rheologies. In every instance where these fluids had been previously studied (including power-law, Carreau, Wyart-Cates, and Herschel-Bulkley fluids), our results exhibited very good agreement with previous studies and experimental findings. Moreover, we successfully predicted precise and analytical long-wave characteristics of roll waves in cases where this had never been accomplished before. For instance, we provided analytical expressions for the threshold and wave celerity of Eyring-Powell fluids, as well as for all regularized rheological laws. Previously, it was believed impossible to derive such analytical expressions for these cases.

Testing our results across a diverse range of fluid rheologies also served to assess the limits and assumptions inherent in the generalized Newtonian rheology. Perhaps the most surprising finding is that, for every singular behavior in the rheology that we examined, the expressions derived from the long-wave analysis still accurately captured the onset of instability, even when the full Orr-Sommerfeld equation was strictly unsolvable. This observation held true when the viscosity diverged (as in power-law or Herschel-Bulkley fluids), as well as when the flow curves exhibited multi-valued behavior (as in Wyart-Cates fluids), even though the instability described in the latter case was inertialess and fundamentally distinct. In the former case, a possible explanation is that these singular rheologies (such as power-law or Herschel-Bulkley fluids) can be viewed as the limit of a series of regularized rheological functions, for which the critical Reynolds number is always well defined and bounded. In the latter case, another explanation could be that if the strain-imposed rheology $\tau (\dot{\gamma })$ is multivalued, the stress-imposed rheology $\dot{\gamma }\left(\tau \right)$ remains well defined, allowing the Orr-Sommerfeld problem to be resolved at any wave number *α*.

Future work should indeed explore the behavior at moderate wavelengths. This investigation could lead to a more precise prediction of the dispersion relation, which would be valuable for providing detailed rheological analyses of fluid behavior. Additionally, it would be intriguing to extend the current model to include rheologies that do not fall within the generalized Newtonian family. For example, this could involve investigating frictional rheology (such as in granular materials), time-dependent rejuvenation (or thixotropy), or even viscoelastic behavior. Another straightforward extension of this work could be to consider the interaction of these roll waves with more complex geometries and physical effects (porous substrate, wavy bottom, thermal gradients or surfactants). Such studies performed with a generalized Newtonian rheology would hopefully produce an expression of the critical Reynolds number and wave celerity similar to those obtained in the present work, but with some additional terms quantifying the relative importance of rheology over these new physical effects. The complex behaviour already observed in power-law fluids [40, 41, 44] suggests however that this may not be a small endeavour, and would warrant several comprehensive studies.

These extensions would enable a more comprehensive understanding of fluid dynamics across a broader range of materials and conditions.

## Supporting information

S1 File Stability analysis calculation details. The S1 File file provides the details of the linear stability analysis that was conducted in this study.
(PDF)

S1 TableDifferent expressions of the critical Reynolds number ${\text{Re}}_{c}^{\theta }$.The S1 Table gives the expression of the critical Reynolds number ${\text{Re}}_{c}^{\theta }$ based on different quantities defined in the present study.(PDF)

S1 Fig Power law model. (PDF)

S2 Fig Carreau model. (PDF)

S3 Fig Ellis model. (PDF)

S4 Fig Eyring Powell model. (PDF)

S5 Fig Wyart-Cates model. (PDF)

S6 Fig Herschel-Bulkley model. (PDF)

S7 Fig Williamson model. (PDF)

S8 FigPapanastasiou model.All these figures show the expressions of dimensionless shear stress $\hat{\tau }$, viscosity $\hat{\eta }$, fluidity $\hat{\Phi }$, base flow $\hat{\bar{u}}$ and critical Reynolds number ${\text{Re}}_{c}^{\theta }$ in their analytical form when it exists. Otherwise it is computed numerically. In all cases, these quantities are plotted as a function of the model rheological parameters.(PDF)

## References

[1] Alekseenko S, Nakoryakov V, Pokusaev B. Wave effect on the transfer processes in liquid films. Chemical Engineering Communications. 1996;141(1):359–385. doi: 10.1080/00986449608936424

[2] Park C, Nosoko T, Gima S, Ro S. Wave-augmented mass transfer in a liquid film falling inside a vertical tube. International Journal of Heat And Mass Transfer. 2004;47(12-13):2587–2598. doi: 10.1016/j.ijheatmasstransfer.2003.12.017

[3] Schweizer PM, Kistler SF. Liquid Film Coating: Scientific principles and their technological implications. Springer Netherlands; 2012. Available from: https://books.google.fr/books?id=5VbtCAAAQBAJ.

[4] Arai M, Huebl J, Kaitna R. Occurrence conditions of roll waves for three grain–fluid models and comparison with results from experiments and field observation. Geophysical Journal International. 2013;195(3):1464–1480. doi: 10.1093/gji/ggt352

[5] Sisoev GM, Dandapat BS, Matveyev KS, Mukhopadhyay A. Bifurcation analysis of the travelling waves on a falling power-law fluid film. Journal of Non-Newtonian Fluid Mechanics. 2007;141(2):128–137. doi: 10.1016/j.jnnfm.2006.09.004

[6] Chang HC, Demekhin E, Kopelevich D. Nonlinear evolution of waves on a vertically falling film. Journal of Fluid Mechanics. 1993;250:433–480. doi: 10.1017/S0022112093001521

[7] Liu J, Schneider J, Gollub JP. Three-dimensional instabilities of film flows. Physics of Fluids. 1995;7(1):55–67. doi: 10.1063/1.868782

[8] Smith MK. The mechanism for the long-wave instability in thin liquid films. Journal of Fluid Mechanics. 1990;217:469–485. doi: 10.1017/S0022112090000805

[9] Kapitza PL. Wave flow of thin layers of viscous liquids. II. Flow in a contact with a Gase flux and Heat transfer. Zhurnal Eksperimentalnoi i Teoreticheskoi Fiziki. 1948;18:19–28.

[10] Benjamin TB. Wave formation in laminar flow down an inclined plane. Journal of Fluid Mechanics. 1957;2(6):554–573. doi: 10.1017/S0022112057000373

[11] Yih C. Stability of Liquid Flow down an Inclined Plane. The Physics of Fluids. 1963;6(3):321–334. doi: 10.1063/1.1706737

[12] Schmid PJ, Henningson DS. Stability and Transition in Shear Flows. Applied Mathematical Sciences. Springer New York; 2000. Available from: https://books.google.fr/books?id=5eNoy2VdXo8C.

[13] Liu J, Paul JD, Gollub JP. Measurements of the primary instabilities of film flows. Journal of Fluid Mechanics. 1993;250:69–101. doi: 10.1017/S0022112093001387

[14] Benney DJ. Long Waves on Liquid Films. Journal of Mathematics and Physics. 1966;45(1-4):150–155. doi: 10.1002/sapm1966451150

[15] Shkadov VY. Wave flow regimes of a thin layer of viscous fluid subject to gravity. Fluid Dynamics. 1967;2(1):29–34. doi: 10.1007/BF01024797

[16] Ruyer-Quil C, Manneville P. Modeling film flows down inclined planes. The European Physical Journal B—Condensed Matter and Complex Systems. 1998;6(2):277–292. doi: 10.1007/s100510050550

[17] Ruyer-Quil C, Manneville P. Improved modeling of flows down inclined planes. The European Physical Journal B—Condensed Matter and Complex Systems. 2000;15(2):357–369. doi: 10.1007/s100510051137

[18] Freydier P, Chambon G, Naaim M. Internal dynamics of a free-surface viscoplastic flow down an inclined channel. International Journal of Erosion Control Engineering. 2016;9(3):101–106. doi: 10.13101/ijece.9.101

[19] De Waele A. Viscometry and plastometry. Oil Color Chem Assoc J. 1923;6:33–88.

[20] Ostwald W. Ueber die Geschwindigkeitsfunktion der Viskosität disperser Systeme. I. Kolloid-Zeitschrift. 1925;36(2):99–117. doi: 10.1007/BF01431449

[21] Ng CO, Mei CC. Roll waves on a shallow layer of mud modelled as a power-law fluid. Journal of Fluid Mechanics. 1994;263:151–184. doi: 10.1017/S0022112094004064

[22] Hwang CC, Chen JL, Wang JS, Lin JS. Linear stability of power law liquid film flows down an inclined plane. Journal of Physics D: Applied Physics. 1994;27(11):2297. doi: 10.1088/0022-3727/27/11/008

[23] Dandapat BS, Mukhopadhyay A. Waves on a film of power-law fluid flowing down an inclined plane at moderate Reynolds number. Fluid Dynamics Research. 2001;29(3):199. doi: 10.1016/S0169-5983(01)00024-7

[24] Dandapat B, Mukhopadhyay A. Waves on the surface of a falling power-law fluid film. International journal of non-linear mechanics. 2003;38(1):21–38. doi: 10.1016/S0020-7462(01)00038-5

[25] Chesnokov A. Formation and evolution of roll waves in a shallow free surface flow of a power-law fluid down an inclined plane. Wave Motion. 2021;106:102799. doi: 10.1016/j.wavemoti.2021.102799

[26] Ruyer-Quil C, Chakraborty S, Dandapat BS. Wavy regime of a power-law film flow. Journal of Fluid Mechanics. 2012;692:220–256. doi: 10.1017/jfm.2011.508

[27] Amaouche M, Djema A, Ait Abderrahmane H. Film flow for power-law fluids: Modeling and linear stability. European Journal of Mechanics—B/Fluids. 2012;34:70–84. doi: 10.1016/j.euromechflu.2012.02.001

[28] Rousset F, Millet S, Botton V, Ben Hadid H. Temporal Stability of Carreau Fluid Flow Down an Incline. Journal of Fluids Engineering. 2007;129(7):913–920. doi: 10.1115/1.2742737

[29] Allouche MH, Botton V, Millet S, Henry D, Dagois-Bohy S, Güzel B, et al. Primary instability of a shear-thinning film flow down an incline: experimental study. Journal of Fluid Mechanics. 2017;821. doi: 10.1017/jfm.2017.276

[30] Balmforth NJ, Liu JJ. Roll waves in mud. Journal of Fluid Mechanics. 2004;519:33–54. doi: 10.1017/S0022112004000801

[31] Noma DM, Dagois-Bohy S, Millet S, Botton V, Henry D, Hadid HB. Primary instability of a visco-plastic film down an inclined plane: experimental study. Journal of Fluid Mechanics. 2021;922. doi: 10.1017/jfm.2021.528

[32] Fernández-Nieto ED, Noble P, Vila JP. Shallow water equations for power law and Bingham fluids. Science China Mathematics. 2012;55(2):277–283. doi: 10.1007/s11425-011-4358-7

[33] de Freitas Maciel G, de Oliveira Ferreira F, Fiorot GH. Control of instabilities in non-Newtonian free surface fluid flows. Journal of the Brazilian Society of Mechanical Sciences and Engineering. 2013;35(3):217–229. doi: 10.1007/s40430-013-0025-y

[34] Calusi B, Farina A, Fusi L, Rosso F. Long-wave instability of a regularized Bingham flow down an incline. Physics of Fluids. 2022;34(5):054111. doi: 10.1063/5.0091260

[35] Calusi B, Farina A, Fusi L, Palade LI. Stability of a Regularized Casson Flow down an Incline: Comparison with the Bingham Case. Fluids. 2022;7(12):380. doi: 10.3390/fluids7120380

[36] Forterre Y, Pouliquen O. Long-surface-wave instability in dense granular flows. Journal of Fluid Mechanics. 2003;486:21–50. doi: 10.1017/S0022112003004555

[37] Darbois Texier B, Lhuissier H, Forterre Y, Metzger B. Surface-wave instability without inertia in shear-thickening suspensions. Communications Physics. 2020;3(1):232. doi: 10.1038/s42005-020-00500-4

[38] Texier BD, Lhuissier H, Metzger B, Forterre Y. Shear-thickening suspensions down inclines: from Kapitza to Oobleck waves. Journal of Fluid Mechanics. 2023;959:A27. doi: 10.1017/jfm.2023.162

[39] Glade RC, Fratkin MM, Pouragha M, Seiphoori A, Rowland JC. Arctic soil patterns analogous to fluid instabilities. Proceedings of the National Academy of Sciences. 2021;118(21):e2101255118. doi: 10.1073/pnas.2101255118 34021079 PMC8166060

[40] Iervolino M, Pascal JP, Vacca A. Instabilities of a power–law film over an inclined permeable plane: A two–sided model. Journal of Non-Newtonian Fluid Mechanics. 2018;259:111–124. doi: 10.1016/j.jnnfm.2018.03.011

[41] Pascal JP, Vacca A. Instabilities of a shear-thinning fluid falling over an undulating porous layer. Journal of Non-Newtonian Fluid Mechanics. 2021;298:104693. doi: 10.1016/j.jnnfm.2021.104693

[42] Hossain MM, Ghosh S, Behera H. Linear instability of a surfactant-laden shear imposed falling film over an inclined porous bed. Physics of Fluids. 2022;34(8). doi: 10.1063/5.0220016

[43] Paul D, Hossain MM, Behera H. Hydrodynamic stability analysis of shear-layered fluid flow over a porous bed in the presence of a floating elastic plate. International Journal of Non-Linear Mechanics. 2024;159:104599. doi: 10.1016/j.ijnonlinmec.2023.104599

[44] Pascal JP, Vacca A. Long-wave instabilities of a power-law fluid flowing over a heated, uneven and porous incline: A two-sided model. Journal of Non-Newtonian Fluid Mechanics. 2024;329:105260. doi: 10.1016/j.jnnfm.2024.105260

[45] Huilgol RR. Fluid Mechanics of Viscoplasticity. Springer Berlin Heidelberg; 2015.

[46] Wilkinson WL. Non-Newtonian fluids: fluid mechanics, mixing and heat transfer. vol. 1. Pergamon; 1960.

[47] Bird RB, Armstrong RC, Hassager O. Dynamics of polymeric liquids. Vol. 1: Fluid mechanics. John Wiley and Sons Inc., New York, NY; 1987.

[48] Carreau PJ, De Kee DC, Chhabra RP. Rheology of polymeric systems: principles and applications. Carl Hanser Verlag GmbH Co KG; 2021.

[49] Tanner RI. Engineering rheology. vol. 52. OUP Oxford; 2000.

[50] Millet S, Botton V, Rousset F, Ben Hadid H. Wave celerity on a shear-thinning fluid film flowing down an incline. Physics of fluids. 2008;20(3). doi: 10.1063/1.2889140

[51] Reiner M, Leaderman H. Deformation, strain, and flow. Physics Today. 1960;13(9):47. doi: 10.1063/1.3057119

[52] Matsuhisa S, Bird RB. Analytical and numerical solutions for laminar flow of the non-Newtonian ellis fluid. AIChE Journal. 1965;11(4):588–595. doi: 10.1002/aic.690110407

[53] Kincaid JF, Eyring H, Stearn AE. The Theory of Absolute Reaction Rates and its Application to Viscosity and Diffusion in the Liquid State. Chemical reviews. 1941;28(2):301–365. doi: 10.1021/cr60090a005

[54] Ree F, Ree T, Eyring H. Relaxation theory of transport problems in condensed systems. Industrial & Engineering Chemistry. 1958;50(7):1036–1040. doi: 10.1021/ie50583a038

[55] Mari R, Seto R, Morris JF, Denn MM. Discontinuous shear thickening in Brownian suspensions by dynamic simulation. Proceedings of the National Academy of Sciences. 2015;112(50):15326–15330. doi: 10.1073/pnas.1515477112 26621744 PMC4687578

[56] Wyart M, Cates ME. Discontinuous Shear Thickening without Inertia in Dense Non-Brownian Suspensions. Physical review letters. 2014;112(9). doi: 10.1103/PhysRevLett.112.098302 24655284

[57] Williamson RV. The flow of pseudoplastic materials. Industrial & Engineering Chemistry. 1929;21(11):1108–1111. doi: 10.1021/ie50239a035

[58] Papanastasiou TC. Flows of Materials with Yield. Journal of Rheology. 1987;31(5):385–404. doi: 10.1122/1.549926

