# Supplementary material for: Unifying the roll waves
Source: PLoS One. 2024 Nov 19;19(11):e0310805. doi: 10.1371/journal.pone.0310805 (PMC11575793; doi:10.1371/journal.pone.0310805)
Supplement: S1 File — The S1 File file provides the details of the linear stability analysis that was conducted in this study. (PDF) [file pone.0310805.s001.pdf]

S1 File. Stability analysis calculation details.

In the following sections, we will provide a comprehensive breakdown of the various steps and calculations summarized in the main paper. Additionally, we will present fluid data sheets to consolidate the key findings of the paper. Specifically, we will focus on elucidating the evolution of the critical Reynolds number as a function of the rheological parameters, encompassing the most prevalent rheological laws encountered in the literature.

Stability Analysis for a Generalized Newtonian Fluid

Rheological Laws and Stress Perturbations

Using the decomposition given in the paper (Eq. 30), the dimensionless shear rate stress tensors become:

$$\hat{\dot{\boldsymbol{\gamma}}} = \begin{pmatrix} 2\partial_{\hat{x}}\tilde{u} & \partial_{\hat{y}}\hat{\bar{u}} + (\partial_{\hat{y}}\tilde{u} + \partial_{\hat{x}}\tilde{v}) \\ \partial_{\hat{y}}\hat{\bar{u}} + (\partial_{\hat{y}}\tilde{u} + \partial_{\hat{x}}\tilde{v}) & -2\partial_{\hat{x}}\tilde{u} \end{pmatrix}, \quad \hat{\boldsymbol{\tau}} = \begin{pmatrix} \tilde{\tau}_{xx} & \hat{\bar{\tau}}_{xy} + \tilde{\tau}_{xy} \\ \hat{\bar{\tau}}_{xy} + \tilde{\tau}_{xy} & -\tilde{\tau}_{xx} \end{pmatrix}.$$

Note that we have used the mass conservation (Eq. 7) in order to write $\partial_y \tilde{v} = -\partial_x \tilde{u}$ in the shear rate tensor. This imposes $\tilde{\tau}_{yy} = -\tilde{\tau}_{xx}$. The shear rate invariant becomes

$$\hat{\dot{\gamma}} = \sqrt{4\left(\partial_{\hat{x}}\tilde{u}\right)^2 + \left(\partial_{\hat{y}}\hat{\bar{u}} + (\partial_{\hat{y}}\tilde{u} + \partial_{\hat{x}}\tilde{v})\right)^2},$$

which after a Taylor expansion and neglecting the quadratic terms in perturbations simplifies into

$$\hat{\dot{\gamma}} = \partial_{\hat{y}}\hat{\bar{u}} + (\partial_{\hat{y}}\tilde{u} + \partial_{\hat{x}}\tilde{v}).$$

In a similar way, the linearised shear stress invariant writes:

$$\hat{\tau} = \hat{\bar{\tau}}_{xy} + \tilde{\tau}_{xy}.$$

Using the dimensionless fluidity definition (Eq. 22), the perturbed rheological equations are

$$2\partial_{\hat{x}}\tilde{u} = \hat{\Phi}\left(\hat{\bar{\tau}}_{xy} + \tilde{\tau}_{xy}\right)\tilde{\tau}_{xx} \quad \text{and} \quad \partial_{\hat{y}}\hat{\bar{u}} + (\partial_{\hat{y}}\tilde{u} + \partial_{\hat{x}}\tilde{v}) = \hat{\Phi}\left(\hat{\bar{\tau}}_{xy} + \tilde{\tau}_{xy}\right)\left(\hat{\bar{\tau}}_{xy} + \tilde{\tau}_{xy}\right),$$

which becomes after linearization

$$2\partial_{\hat{x}}\tilde{u} = \hat{\Phi}\left(\hat{\bar{\tau}}_{xy}\right)\tilde{\tau}_{xx} \quad \text{and} \quad \partial_{\hat{y}}\tilde{u} + \partial_{\hat{x}}\tilde{v} = \left(\hat{\Phi}\left(\hat{\bar{\tau}}_{xy}\right) + \hat{\bar{\tau}}_{xy}\frac{d\hat{\Phi}}{d\hat{\tau}}\left(\hat{\bar{\tau}}_{xy}\right)\right)\tilde{\tau}_{xy},$$

as we know that $\partial_{\hat{y}}\hat{\bar{u}} = \hat{\Phi}\left(\hat{\bar{\tau}}_{xy}\right)\hat{\bar{\tau}}_{xy}$.

The stress perturbations can then be written introducing the functions δ and γ (see Eqs. 31-32):

$$\tilde{\tau}_{xx} = 2\delta\partial_{\hat{x}}\tilde{u}, \quad \text{and} \quad \tilde{\tau}_{xy} = \gamma\left(\partial_{\hat{y}}\tilde{u} + \partial_{\hat{x}}\tilde{v}\right),$$

with

$$\delta(\hat{y}) = \frac{1}{\hat{\Phi}\left(\hat{\bar{\tau}}_{xy}\right)} = \frac{1}{\hat{\Phi}(1-\hat{y})} = \frac{1-\hat{y}}{\hat{\bar{u}}'(\hat{y})},$$

and

$$\gamma(\hat{y}) = \frac{1}{\hat{\Phi}\left(\hat{\bar{\tau}}_{xy}\right) + \hat{\bar{\tau}}_{xy}\frac{d\hat{\Phi}}{d\hat{\tau}}\left(\hat{\bar{\tau}}_{xy}\right)} = \frac{1}{\hat{\Phi}(1-\hat{y}) + (1-\hat{y})\frac{d\hat{\Phi}}{d\hat{\tau}}(1-\hat{y})} = \frac{-1}{\hat{\bar{u}}''(\hat{y})}.$$

Mass Conservation and Momentum Balance Equations

Once projected on the x and y axis, Eqs. 7 and 8 write:

$$\partial_x u + \partial_y v = 0,$$

$$\begin{cases} \rho\left(\partial_t u + u\partial_x u + v\partial_y u\right) = -\partial_x p + \partial_x \tau_{xx} + \partial_y \tau_{xy} + \rho g \sin\left(\theta\right), \\ \rho\left(\partial_t v + u\partial_x v + v\partial_y v\right) = -\partial_y p + \partial_x \tau_{xy} + \partial_y \tau_{yy} - \rho g \cos\left(\theta\right), \end{cases}$$

where ∂ are partial derivatives and $\tau_{xx} = 2\eta\left(\dot{\gamma}\right)\partial_x u$, $\tau_{xy} = \eta\left(\dot{\gamma}\right)\left(\partial_y u + \partial_x v\right)$, $\tau_{yy} = 2\eta\left(\dot{\gamma}\right)\partial_y v$.

These equations are given in their dimensionless form in Eqs. 27-29.

We introduce the decomposition (Eq. 30) in the equations of motion. After neglecting quadratic terms in perturbations and removing all terms appearing in the base flow equations, we obtain:

$$\begin{cases} \mathrm{Re}\left(\partial_{\hat{t}}\tilde{u} + \hat{\bar{u}}\partial_{\hat{x}}\tilde{u} + \tilde{v}\partial_{\hat{y}}\hat{\bar{u}}\right) & = -\mathrm{Re}\,\partial_{\hat{x}}\tilde{p} + \partial_{\hat{x}}\tilde{\tau}_{xx} + \partial_{\hat{y}}\tilde{\tau}_{xy}, \\ \mathrm{Re}\left(\partial_{\hat{t}}\tilde{v} + \hat{\bar{u}}\partial_{\hat{x}}\tilde{v}\right) & = -\mathrm{Re}\,\partial_{\hat{y}}\tilde{p} + \partial_{\hat{x}}\tilde{\tau}_{xy} - \partial_{\hat{y}}\tilde{\tau}_{xx}. \end{cases}$$

After replacing $\tilde{\tau}_{xx}$ and $\tilde{\tau}_{xy}$ with their expression given in Eq. 31, the problem can be expressed as

$$\begin{cases} \mathrm{Re}\left(\partial_{\hat{t}}\tilde{u} + \hat{\bar{u}}\partial_{\hat{x}}\tilde{u} + \tilde{v}\partial_{\hat{y}}\hat{\bar{u}}\right) & = -\mathrm{Re}\,\partial_{\hat{x}}\tilde{p} + 2\delta\partial_{xx}\tilde{u} + \partial_{\hat{y}}\left(\gamma\left(\partial_{\hat{y}}\tilde{u} + \partial_{\hat{x}}\tilde{v}\right)\right), \\ \mathrm{Re}\left(\partial_{\hat{t}}\tilde{v} + \hat{\bar{u}}\partial_{\hat{x}}\tilde{v}\right) & = -\mathrm{Re}\,\partial_{\hat{y}}\tilde{p} + \gamma\partial_{\hat{x}}\left(\partial_{\hat{y}}\tilde{u} + \partial_{\hat{x}}\tilde{v}\right) - 2\partial_{\hat{y}}\left(\delta\partial_{\hat{x}}\tilde{u}\right). \end{cases} \quad \text{(A-1)}$$

The next step towards the generalised Orr-Sommerfeld equation is the elimination of the pressure terms, applying the curl operator to the previous system:

$$\mathrm{Re}\left(\partial_{\hat{t}\hat{x}}\tilde{v} + \hat{\bar{u}}\partial_{\hat{x}\hat{x}}\tilde{v} - \partial_{\hat{t}\hat{y}}\tilde{u} - \partial_{\hat{y}}\left(\hat{\bar{u}}\partial_{\hat{x}}\tilde{u}\right) - \partial_{\hat{y}}\left(\tilde{v}\partial_{\hat{y}}\hat{\bar{u}}\right)\right) = \gamma\partial_{\hat{x}\hat{x}}\left(\partial_{\hat{y}}\tilde{u} + \partial_{\hat{x}}\tilde{v}\right) - 4\partial_{\hat{y}}\left(\delta\partial_{\hat{x}\hat{x}}\tilde{u}\right) - \partial_{\hat{y}\hat{y}}\left(\gamma\left(\partial_{\hat{y}}\tilde{u} + \partial_{\hat{x}}\tilde{v}\right)\right).$$

We then introduce a stream function Ψ for the perturbation (Eq. 33) and it becomes

$$\mathrm{Re}\left(-\partial_{\hat{t}\hat{x}\hat{x}}\Psi - \hat{\bar{u}}\partial_{\hat{x}\hat{x}\hat{x}}\Psi - \partial_{tyy}\Psi - \partial_{\hat{y}}\left(\hat{\bar{u}}\partial_{xy}\Psi\right) + \partial_{\hat{y}}\left(\partial_{\hat{x}}\Psi\partial_{\hat{y}}\hat{\bar{u}}\right)\right) = \gamma\partial_{\hat{x}\hat{x}}\left(\partial_{\hat{y}\hat{y}}\Psi - \partial_{\hat{x}\hat{x}}\Psi\right) - 4\partial_{\hat{y}}\left(\delta\partial_{\hat{x}\hat{x}\hat{y}}\Psi\right) - \partial_{\hat{y}\hat{y}}\left(\gamma\left(\partial_{\hat{y}\hat{y}}\Psi - \partial_{\hat{x}\hat{x}}\Psi\right)\right),$$

and leads to Eq. 35 when substituting Ψ with its decomposition into normal modes.

Boundary Conditions at the Free Surface

The outward normal vector $\boldsymbol{n}$ to the free surface writes $(1+\partial_x h^2)^{-1/2}\begin{pmatrix} -\partial_x h \\ 1 \end{pmatrix}$ in the (x, y) axis system. Projecting Eq. 14 yields two equations:

$$\begin{cases} -\left(\tau_{xx} - p\right)\partial_x h + \tau_{xy} = \left(p_0 - 2HS\right)\partial_x h, \\ -\tau_{xy}\partial_x h + \tau_{yy} - p = -p_0 + 2HS, \end{cases} \quad \text{at} \quad y = h\left(x, t\right).$$

The mean curvature is given by $H = \dfrac{\partial_{xx} h}{2\left(1 + \left(\partial_x h\right)^2\right)^{3/2}}$. It is fairly common to project these two equations along the normal and tangent vectors. The previous system then becomes:

$$\begin{cases} p_0 - p + \left(\tau_{xx}\left(\partial_x h\right)^2 - 2\tau_{xy}\partial_x h + \tau_{yy}\right)\left(1 + \left(\partial_x h\right)^2\right)^{-1} = S\partial_{xx} h\left(1 + \left(\partial_x h\right)^2\right)^{-3/2}, \\ \tau_{xy}\left(1 - \left(\partial_x h\right)^2\right) - \left(\tau_{xx} - \tau_{yy}\right)\partial_x h = 0, \end{cases} \quad \text{at} \quad y = h\left(x, t\right).$$

The dimensionless boundary conditions at the free surface are then:

$$\begin{cases} \mathrm{Re}\left(\hat{p}_0 - \hat{p}\right) + \left(\hat{\tau}_{xx}\left(\partial_{\hat{x}}\hat{h}\right)^2 - 2\hat{\tau}_{xy}\partial_{\hat{x}}\hat{h} + \hat{\tau}_{yy}\right)\left(1 + \left(\partial_{\hat{x}}\hat{h}\right)^2\right)^{-1} = T\mathrm{Re}\partial_{\hat{x}\hat{x}}\hat{h}\left(1 + \left(\partial_{\hat{x}}\hat{h}\right)^2\right)^{-3/2}, \\ \hat{\tau}_{xy}\left(1 - \left(\partial_{\hat{x}}\hat{h}\right)^2\right) - (\hat{\tau}_{xx} - \hat{\tau}_{yy})\partial_{\hat{x}}\hat{h} = 0, \end{cases} \quad \text{at} \quad \hat{y} = \hat{h},$$

with T a dimensionless number, defined in Eq. 38.
Finally, the kinematic boundary condition in dimensionless form writes:

$$\hat{v} = \partial_{\hat{t}}\hat{h} + \hat{u}\partial_{\hat{x}}\hat{h}, \quad \text{at} \quad \hat{y} = 1.$$

The introduction of the decomposition (Eq. 30) in the free surface and the kinematic boundary conditions gives, at first order in perturbations:

$$\begin{cases} \mathrm{Re}\left(\hat{p}_0 - \hat{\bar{p}} - \tilde{p}\right) - \tilde{\tau}_{xx} & = T\,\mathrm{Re}\,\partial_{\hat{x}\hat{x}}\tilde{h}, \\ \hat{\bar{\tau}}_{xy} + \tilde{\tau}_{xy} & = 0, \\ \partial_{\hat{t}}\tilde{h} + \hat{\bar{u}}\partial_{\hat{x}}\tilde{h} & = \tilde{v}, \end{cases} \quad \text{at} \quad \hat{y} = 1 + \tilde{h}.$$

Since these boundary conditions are expressed at $\hat{y} = 1 + \tilde{h}$, a series expansion of every term is done in order to write them at $\hat{y} = 1$. In particular,

$$\hat{\bar{p}}\left(1 + \tilde{h}\right) = \hat{\bar{p}}\,(1) + \tilde{h}\partial_{\hat{y}}\hat{\bar{p}}\,(1) = \hat{p}_0 - \tilde{h}\,\frac{\cot(\theta)}{\mathrm{Re}},$$
$$\hat{\bar{\tau}}_{xy}\left(1 + \tilde{h}\right) = \hat{\bar{\tau}}_{xy}\,(1) + \tilde{h}\partial_y\hat{\bar{\tau}}_{xy}\,(1) = -\tilde{h}.$$

The perturbed boundary conditions expressed at $\hat{y} = 1$ are then, after substituting $\tilde{\tau}_{xx}$ and $\tilde{\tau}_{xy}$ by their expression (Eq. 31):

$$\begin{cases} \mathrm{Re}\,\tilde{p} + 2\delta\partial_{\hat{x}}\tilde{u} & = \tilde{h}\cot(\theta) - T\mathrm{Re}\partial_{\hat{x}\hat{x}}\tilde{h}, \\ \gamma\left(\partial_{\hat{y}}\tilde{u} + \partial_{\hat{x}}\tilde{v}\right) & = \tilde{h}, \\ \partial_{\hat{t}}\tilde{h} + \hat{\bar{u}}\partial_{\hat{x}}\tilde{h} & = \tilde{v}. \end{cases}$$

To eliminate the pressure in the first equation, it is differentiated with respect to $\hat{x}$ and using the first perturbed equation of motion A-1, we have:

$$\mathrm{Re}\,\partial_{\hat{x}}\tilde{p} = \partial_{\hat{x}}\tilde{h}\cot\theta - T\mathrm{Re}\partial_{\hat{x}\hat{x}\hat{x}}\tilde{h} - 4\delta\partial_{\hat{x}\hat{x}}\tilde{u} = \partial_{\hat{y}}\left(\gamma\left(\partial_{\hat{y}}\tilde{u} + \partial_{\hat{x}}\tilde{v}\right)\right) - \mathrm{Re}\left(\partial_{\hat{t}}\tilde{u} + \hat{\bar{u}}\partial_{\hat{x}}\tilde{u} + \tilde{v}\partial_{\hat{y}}\hat{\bar{u}}\right).$$

Finally, after introducing the stream function (Eq. 33) with the normal modes decomposition (Eq. 34) , the boundary conditions at the free surface are obtained in Eq. 37.

The generalised Orr-Sommerfeld equation (Eq. 35) associated with boundary conditions (Eq. 36) and (Eq. 37) is a generalized eigenvalue problem.

Long Wave Expansion

Substituting the series given in Eq. 39 into the Orr-Sommerfeld system (Eqs. 35-37), and collecting terms to the zeroth-order in α, one has the following differential system:

$$\begin{cases} \left(\gamma\psi_0''\right)'' = 0, \\ \psi_0\,(0) = 0, \\ \psi_0'\,(0) = 0, \\ \gamma\psi_0''\,(1) = \xi, \\ \left(\gamma\psi_0''\right)'\,(1) = 0, \\ \xi(c_0 - \hat{\bar{u}}(1)) = \psi_0(1). \end{cases}$$

The eigenfunction is found to be to $\psi_0\,(\hat{y}) = \xi\left(\hat{y} - \hat{\bar{u}}\,(\hat{y})\right)$, and the eigenvalue is $\hat{c}_0 = 1$ (Eq. 40).

The first-order approximation obtained by collecting terms of order α into the Orr-Sommerfeld system (Eqs. 35-37) gives rise to the following system:

$$\begin{cases} \left(\gamma\psi_1''\right)'' = i\,\mathrm{Re}\left(\left(\hat{\bar{u}} - 1\right)\psi_0'' - \hat{\bar{u}}''\psi_0\right), \\ \psi_1\,(0) = 0, \\ \psi_1'\,(0) = 0, \\ \gamma\psi_1''(1) = 0, \\ \left(\gamma\psi_1''\right)'\,(1) = i\xi\cot(\theta) + i\mathrm{Re}\left(\hat{\bar{u}}(1) - 1\right)\psi_0'(1), \\ \xi c_1 = \psi_1(1). \end{cases}$$

One obtains

$$\psi_1(\hat{y}) = i\xi\,\mathrm{Re}\int_0^{\hat{y}}\int_0^{y_1}(-\hat{\bar{u}}''(y_2))\left[(1-y_2)(\hat{\bar{u}}(y_2)+1)+2\int_1^{y_2}\hat{\bar{u}}(y_3)\,dy_3\right]dy_2\,dy_1 \\ - i\xi\cot(\theta)\int_0^{\hat{y}}\int_0^{y_1}(1-y_2)(-\hat{\bar{u}}''(y_2))\,dy_2\,dy_1,$$

and

$$\hat{c}_1 = i\,\mathrm{Re}\int_0^1\int_0^y(-\hat{\bar{u}}''(y_1))\left[(1-y_1)(\hat{\bar{u}}(y_1)+1)+2\int_1^{y_1}\hat{\bar{u}}(y_2)\,dy_2\right]dy_1 dy - i\cot(\theta)\int_0^1\int_0^y(1-y_1)(-\hat{\bar{u}}''(y_1))dy_1\,dy.$$

Using integration by parts, it is possible to re-arrange this expression. First, we have:

$$\begin{aligned}\int_0^1\int_0^y(1-y_1)(-\hat{\bar{u}}''(y_1))\,dy_1\,dy &= \int_0^1\left\{\left[-\hat{\bar{u}}'(y)(1-y)\right]_0^y+\int_0^y(-\hat{\bar{u}}'(y_1))\,dy_1\right\}dy \\ &= \int_0^1\left\{-\hat{\bar{u}}'(y)(1-y)+1+\int_0^y(-\hat{\bar{u}}'(y_1))\,dy_1\right\}dy \\ &= 1-2\int_0^1\hat{\bar{u}}(y)\,dy = 1-2\hat{q}\ ,\end{aligned}$$

with $\hat{q}=\int_0^1\hat{\bar{u}}(y)dy$ the dimensionless flow rate. Second, we have:

$$\begin{aligned}\int_0^1\int_0^y(-\hat{\bar{u}}''(y_1))\int_1^{y_1}\hat{\bar{u}}(y_2)\,dy_2\,dy_1\,dy &= \int_0^1\int_0^y(-\hat{\bar{u}}''(y_1))\left\{\int_0^{y_1}\hat{\bar{u}}(y_2)\,dy_2+\hat{q}\right\}dy_1\,dy \\ &= \int_0^1\left\{-\hat{\bar{u}}'(y))\int_0^y\hat{\bar{u}}(y_1)\,dy_1+\int_0^y\hat{\bar{u}}'(y_1)\hat{\bar{u}}(y_1)\,dy_1\right\}dy+\hat{q}(\hat{\bar{u}}(1)-1) \\ &= \left[-\hat{\bar{u}}(y))\int_0^y\hat{\bar{u}}(y_1)\,dy_1\right]_0^1+\frac{3}{2}\int_0^1\hat{\bar{u}}^2(y)dy+\hat{q}(\hat{\bar{u}}(1)-1) \\ &= \frac{3}{2}\mathcal{M}-q.\end{aligned}$$

Finally, we have:

$$\begin{aligned}\int_0^1\int_0^y(-\hat{\bar{u}}''(y_1))(1-y_1)(\hat{\bar{u}}(y_1)+1)\,dy_1\,dy &= 1-2\hat{q}+\int_0^1\int_0^y(-\hat{\bar{u}}''(y_1))\hat{\bar{u}}(y_1)(1-y_1)\,dy_1\,dy \\ &= 1-2\hat{q}+\int_0^1\left\{(-\hat{\bar{u}}'(y))\hat{\bar{u}}(y)(1-y)-\int_0^y(-\hat{\bar{u}}'(y_1))\left[\hat{\bar{u}}'(y_1)(1-y_1)-\hat{\bar{u}}(y_1)\right]dy_1\right\} \\ &= 1-2\hat{q}-\frac{1}{2}\int_0^1\hat{\bar{u}}(y)\,dy-\frac{1}{2}\int_0^1\hat{\bar{u}}(y)\,dy+\int_0^1\int_0^y\hat{\bar{u}}'^2(y_1)(1-y_1)\,dy_1\,dy \\ &= 1-2\hat{q}-\mathcal{M}+\int_0^1(1-y)\int_0^y\hat{\bar{u}}'^2(y_1)\,dy_1\,dy+\int_0^1\int_0^y\int_0^{y_1}\hat{\bar{u}}'^2(y_2)\,dy_2\,dy_1\,dy \\ &= 1-2\hat{q}-\mathcal{M}+2\mathcal{K}.\end{aligned}$$

It leads to the expression of the critical Reynolds number given in Eq. 42 solving $\hat{c}_1=0$. The expression of $\hat{c}_1$ can thus be rewritten as Eq. 41 where the pre-factor A is the denominator of Eq 42:

$$A = 1-4\hat{q}+2\mathcal{M}+2\mathcal{K}.$$

The phase velocity $\hat{c}_1$ is a pure imaginary and it is proportional to the growth rate. As long as $\mathrm{Re}_c>0$, the flow is stable, neutrally stable or unstable whether the Reynolds number is respectively less, equal or greater than Re_c. In Eq. 42, the critical Reynolds number only involves the base flow velocity profile $\hat{\bar{u}}$. If needed, it can alternatively be expressed through the fluidity function or through the $\hat{G}$ function. For the convenience of the reader, we give the different expressions of Re_c^θ in file **S1 Table**.

Kinematic Wave Speed

According to Eq 18, the dimensional flow rate is

$$q = \int_0^h \int_0^y G(\rho g S(h - y_1))\, dy_1\, dy,$$

with $S = \sin\theta$. The celerity of kinematic waves is defined as $c^{\mathrm{kin}} = \dfrac{dq}{dh}$, which gives:

$$\begin{aligned} c^{\mathrm{kin}} &= \int_0^h G(\rho g S(h - y_1))\, dy_1 + \int_0^h \int_0^y \rho g S G'(\rho g S(h - y_1))\, dy_1\, dy \\ &= \int_0^h G(\rho g S(h - y_1))\, dy_1 + \int_0^h G(\rho g S h)\, dy - \int_0^h G(\rho g S(h - y))\, dy \\ &= h\dot{\gamma}_b, \end{aligned}$$

with $\dot{\gamma}_b = G(\rho g S h)$ the bottom shear rate.
