# Supplementary material for: Unifying the roll waves
Source: PLoS One. 2024 Nov 19;19(11):e0310805. doi: 10.1371/journal.pone.0310805 (PMC11575793; doi:10.1371/journal.pone.0310805)
Supplement: S1 Table — The S1 Table gives the expression of the critical Reynolds number Recθ based on different quantities defined in the present study. (PDF) [file pone.0310805.s002.pdf]

Expression based on the dimensionless velocity of the base flow calculated through Eq. 26:

$$\mathrm{Re}_c^{\theta} = \frac{\displaystyle\int_0^1 \int_0^{\hat{y}} \hat{\hat{u}}''(y_1)\,(1-y_1)\,dy_1\,d\hat{y}}{\displaystyle\int_0^1 \int_0^{\hat{y}} \left((1-y_1)\left(1+\hat{\hat{u}}(y_1)\right)\hat{\hat{u}}''(y_1) + 2\hat{\hat{u}}''(y_1)\int_1^{y_1} \hat{\hat{u}}(y_2)\,dy_2 \right) dy_1\,d\hat{y}}.$$

Expression based on the $\hat{G}(1-\hat{y})$ function given by Eq. 22:

$$\mathrm{Re}_c^{\theta} = \frac{\displaystyle\int_0^1 \int_0^{\hat{y}} (1-y_1)\,\hat{G}'(1-y_1)\,dy_1\,d\hat{y}}{\displaystyle\int_0^1 \int_0^{\hat{y}} \hat{G}'(1-y_1)\left[(1-y_1)\left(1+\int_0^{y_2} \hat{G}(1-y_2)\,dy_2\right) + 2\int_1^{y_1}\int_0^{y_2} \hat{G}(1-y_3)\,dy_3\,dy_2\right] dy_1\,d\hat{y}}.$$

Expression based on the flow rate, form factor and higher moments of the base flow profile

$$\mathrm{Re}_c^{\theta} = \frac{1-2\hat{q}}{1-4\hat{q}+2\mathcal{M}+2\mathcal{K}},$$

with $\hat{q} = \int_0^1 \hat{\hat{u}}(y)\,dy$ the flow rate, $\mathcal{M} = \int_0^1 \hat{\hat{u}}^2(y)\,dy$ the form factor, and $\mathcal{K} = \int_0^1 \int_0^y \int_0^{y_1} \hat{\hat{u}}'^2 dy_2\,dy_1\,dy$.

S1 Table. Different expressions of the critical Reynolds number Re_c^{θ}.
