# Supplementary material for: Unifying the roll waves
Source: PLoS One. 2024 Nov 19;19(11):e0310805. doi: 10.1371/journal.pone.0310805 (PMC11575793; doi:10.1371/journal.pone.0310805)

Power law

Shear stress: $\hat{\tau}\left(\hat{\dot{\gamma}}\right) = \hat{\dot{\gamma}}^{n}$

Viscosity: $\hat{\eta}\left(\hat{\dot{\gamma}}\right) = \hat{\dot{\gamma}}^{n-1}$

Fluidity: $\hat{\Phi}\left(\hat{\tau}\right) = \hat{\tau}^{\frac{1}{n}-1}$

Base flow: $\hat{u}\left(\hat{y}\right) = \dfrac{1-(1-\hat{y})^{m}}{m}$, with $m = \dfrac{n+1}{n}$

Critical Reynolds: $\mathrm{Re}_c^{\theta} = 1 + \dfrac{3n}{2}$

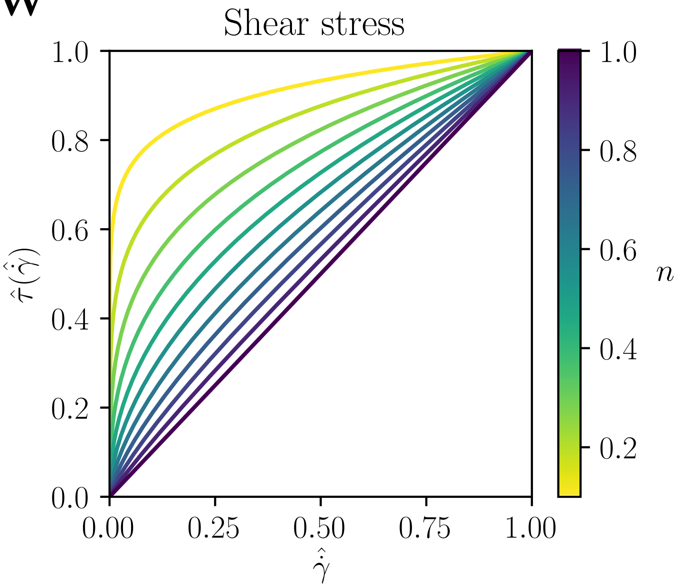

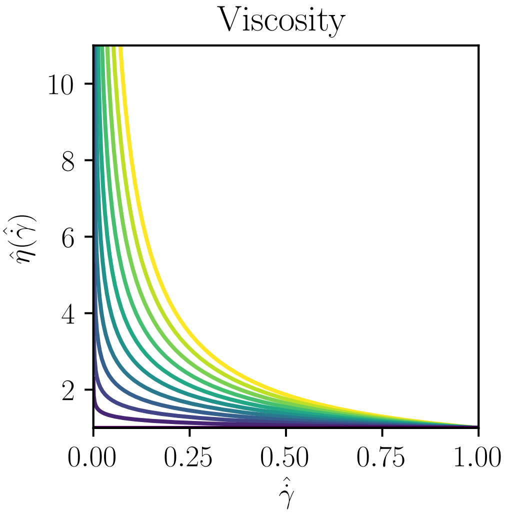

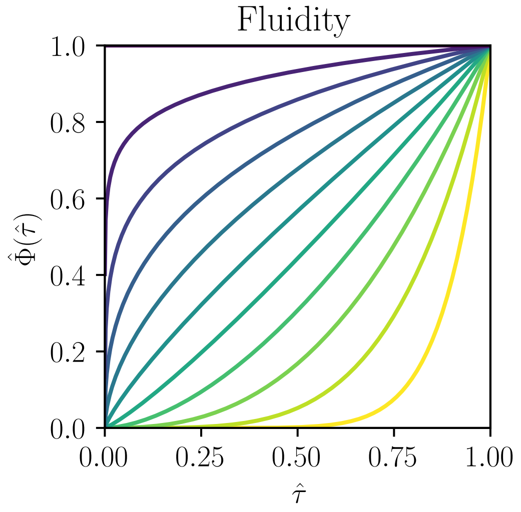

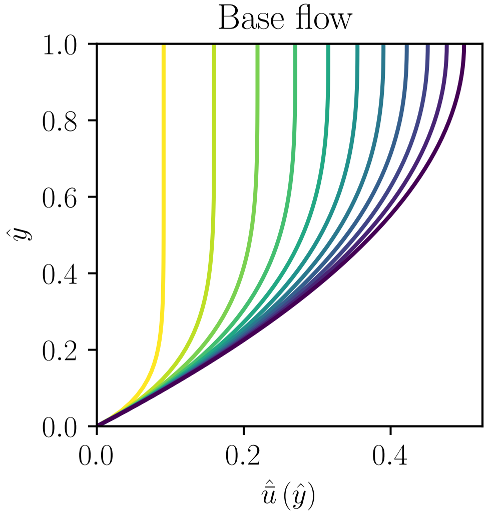

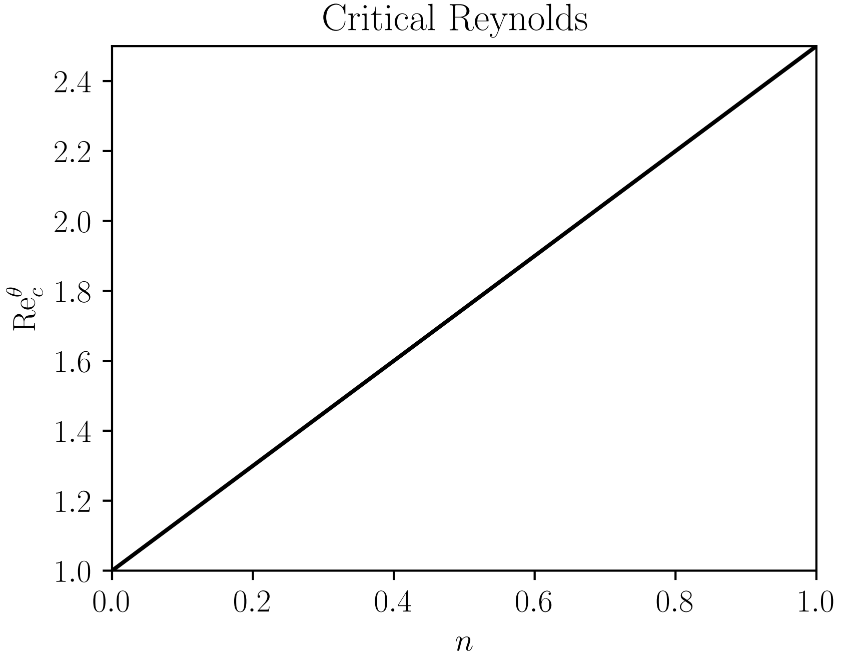

Supplement: S1 Fig — (PDF) [file pone.0310805.s003.pdf]
