# Supplementary material for: Unifying the roll waves
Source: PLoS One. 2024 Nov 19;19(11):e0310805. doi: 10.1371/journal.pone.0310805 (PMC11575793; doi:10.1371/journal.pone.0310805)

Carreau

Shear stress: $\hat{\tau}\left(\hat{\dot{\gamma}}\right) = \left(\dfrac{1+\left(\hat{\dot{\gamma}}/\hat{\dot{\gamma}}_0\right)^2}{1+\left(1/\hat{\dot{\gamma}}_0\right)^2}\right)^{\frac{n-1}{2}} \hat{\dot{\gamma}}$

Viscosity: $\hat{\eta}\left(\hat{\dot{\gamma}}\right) = \left(\dfrac{1+\left(\hat{\dot{\gamma}}/\hat{\dot{\gamma}}_0\right)^2}{1+\left(1/\hat{\dot{\gamma}}_0\right)^2}\right)^{\frac{n-1}{2}}$

Fluidity: $\hat{\Phi}(\hat{\tau})$ numerically computed

Base flow: $\hat{u}(\hat{y})$ numerically computed

Critical Reynolds: Re_c^θ numerically computed

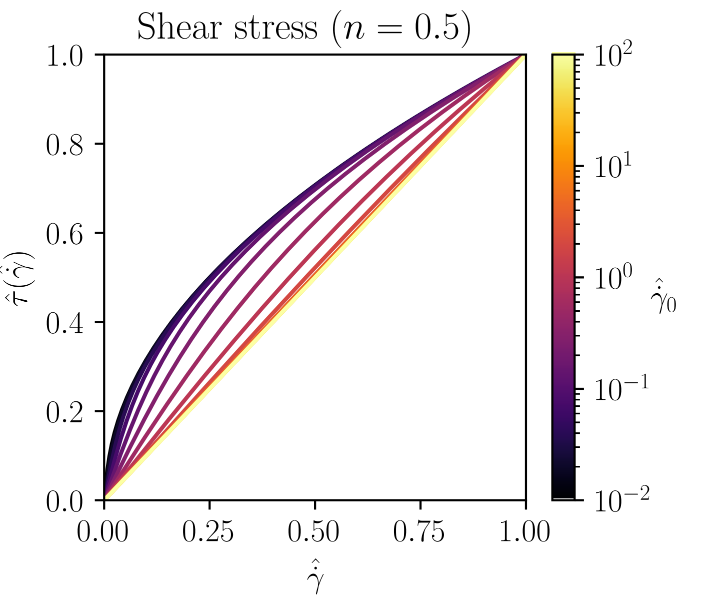

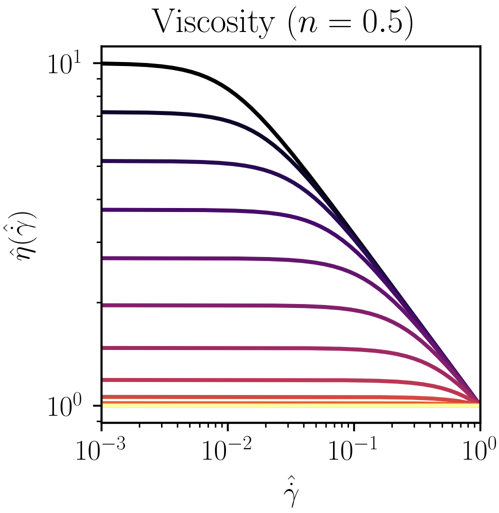

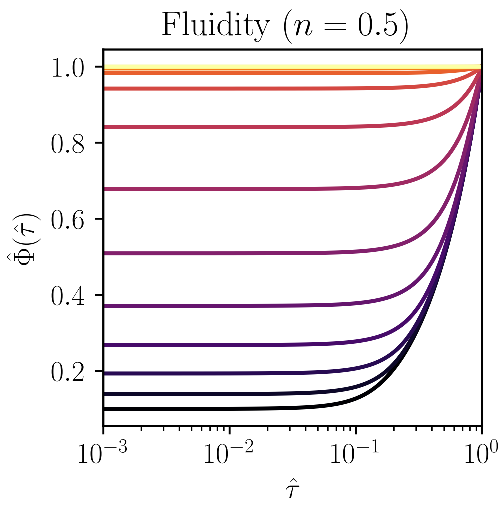

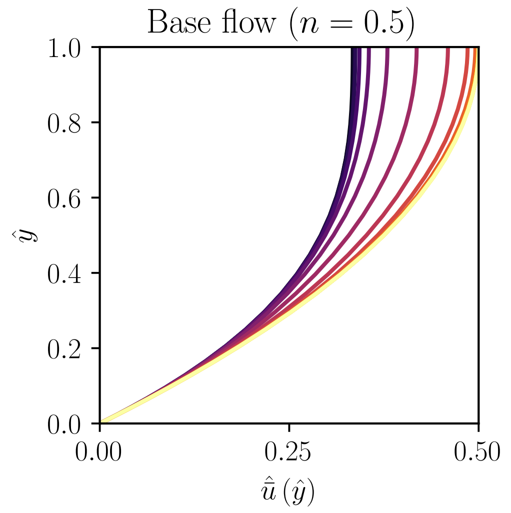

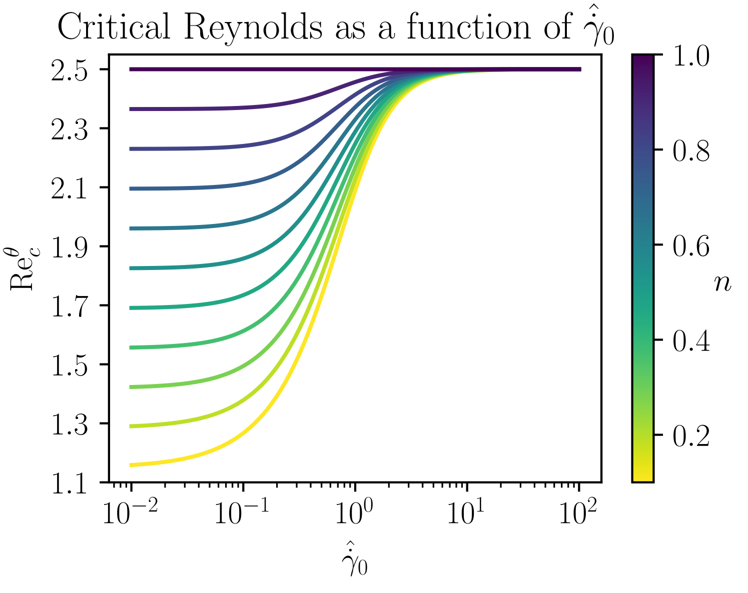

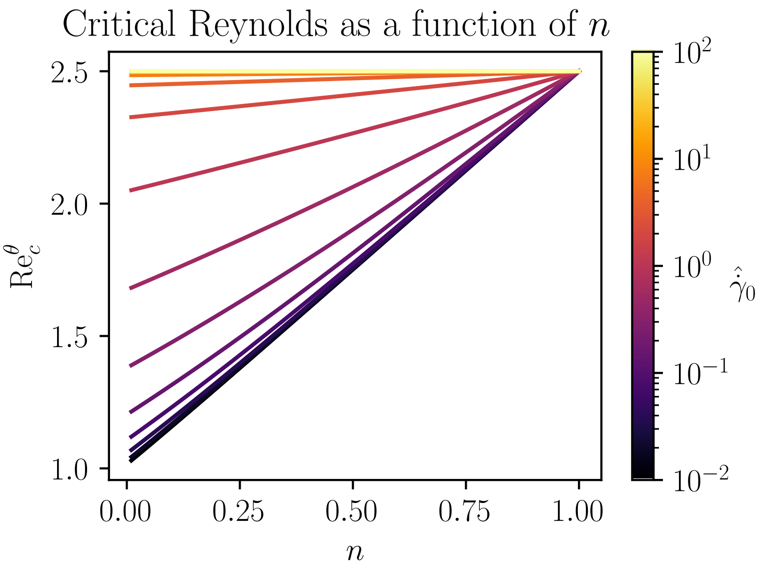

Supplement: S2 Fig — (PDF) [file pone.0310805.s004.pdf]
