# Supplementary material for: Unifying the roll waves
Source: PLoS One. 2024 Nov 19;19(11):e0310805. doi: 10.1371/journal.pone.0310805 (PMC11575793; doi:10.1371/journal.pone.0310805)

Ellis

Shear stress: $\hat{\tau}(\hat{\dot{\gamma}})$ numerically computed

Viscosity: $\hat{\eta}(\hat{\dot{\gamma}})$ numerically computed

Fluidity: $$\hat{\Phi}(\hat{\tau}) = \frac{1+\left(\frac{\hat{\tau}}{\hat{\tau}_{1/2}}\right)^{-1+1/n}}{1+\left(\frac{1}{\hat{\tau}_{1/2}}\right)^{-1+1/n}}$$

Base flow: $$\hat{\bar{u}}(\hat{y}) = \frac{\frac{n}{n+1}\left(1-(1-\hat{y})^{1+1/n}\right)+\hat{\tau}_{1/2}^{-1+1/n}\,(1-\hat{y}/2)\,\hat{y}}{1+\hat{\tau}_{1/2}^{-1+1/n}}$$

Critical Reynolds: $$\mathrm{Re}_c^{\theta} = \frac{5\left(1+\hat{\tau}_{1/2}^{-1+1/n}\right)\left(1+2n+3\hat{\tau}_{1/2}^{1-1/n}\right)}{\frac{30}{2+3n}\hat{\tau}_{1/2}^{2-2/n}+2\,(1+2n)+\frac{10(1+5n)}{1+4n}\hat{\tau}_{1/2}^{1-1/n}}$$

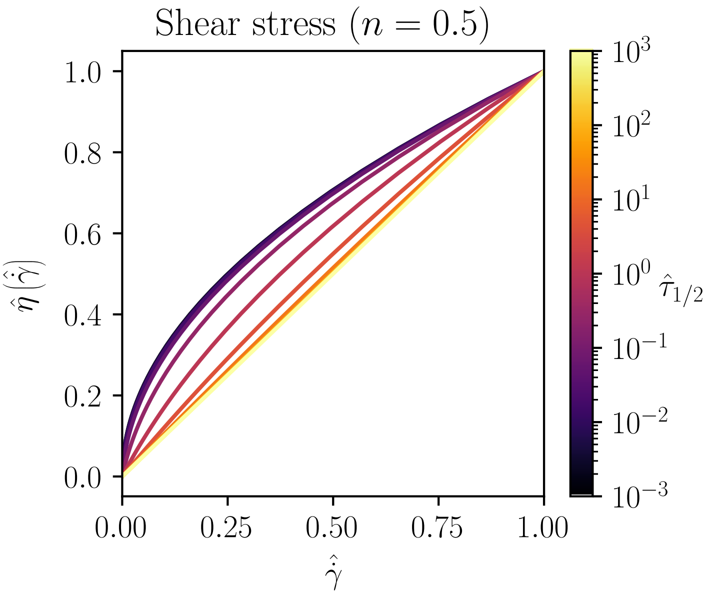

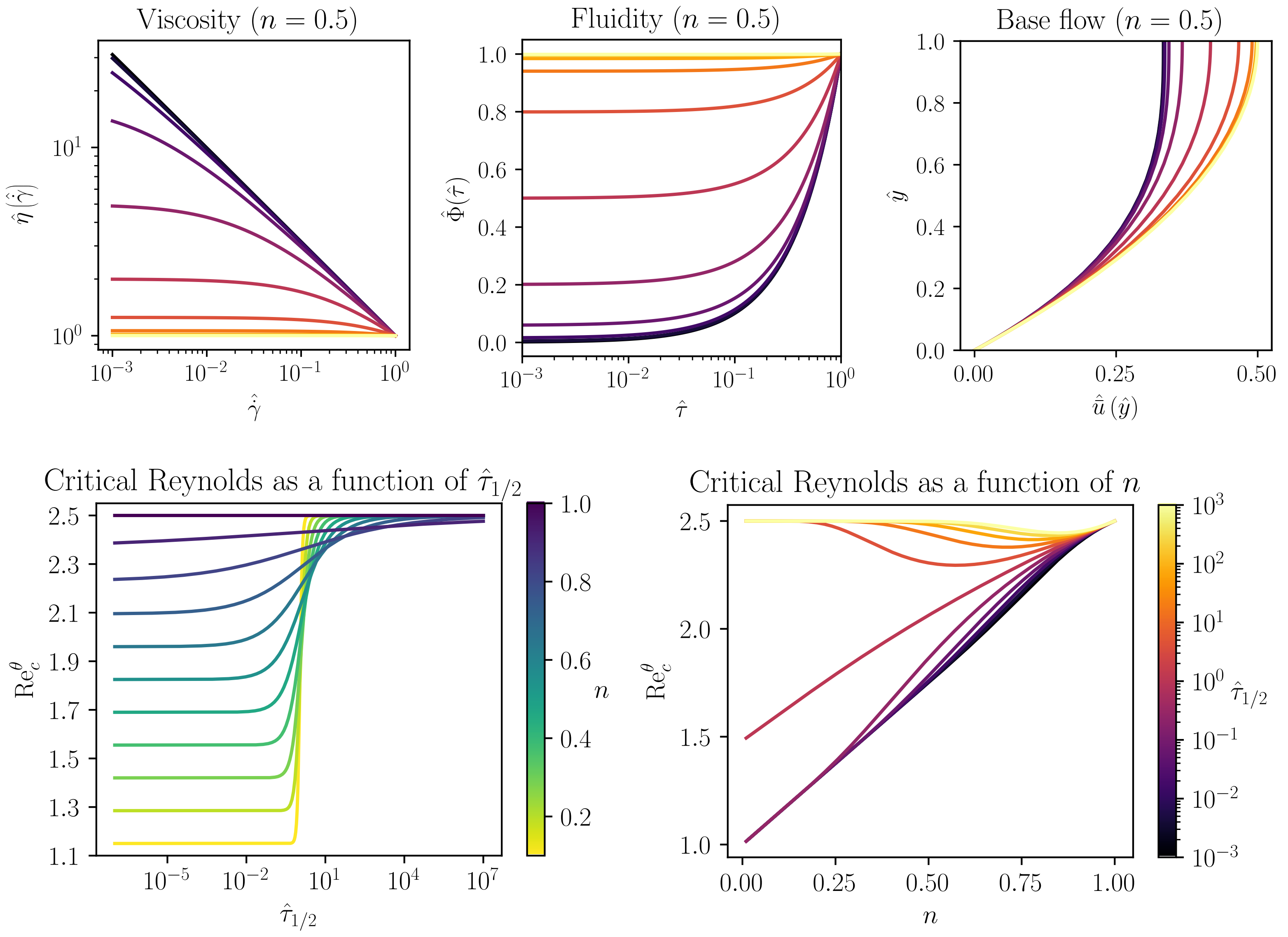

Supplement: S3 Fig — (PDF) [file pone.0310805.s005.pdf]
