# Supplementary material for: Unifying the roll waves
Source: PLoS One. 2024 Nov 19;19(11):e0310805. doi: 10.1371/journal.pone.0310805 (PMC11575793; doi:10.1371/journal.pone.0310805)

Eyring Powell

Shear stress: $\hat{\tau}\left(\hat{\dot{\gamma}}\right) = \dfrac{\operatorname{asinh}\left(\sinh\left(\hat{\delta}\right)\hat{\dot{\gamma}}\right)}{\hat{\delta}}$, with $\hat{\delta} = \delta\dot{\gamma}_b$

Viscosity: $\hat{\eta}\left(\hat{\dot{\gamma}}\right) = \dfrac{\operatorname{asinh}\left(\sinh\left(\hat{\delta}\right)\hat{\dot{\gamma}}\right)}{\hat{\delta}\hat{\dot{\gamma}}}$

Fluidity: $\hat{\Phi}\left(\hat{\tau}\right) = \dfrac{\sinh\left(\hat{\delta}\hat{\tau}\right)}{\sinh\left(\hat{\delta}\right)\hat{\tau}}$

Base flow: $\hat{u}\left(\hat{y}\right) = \dfrac{\cosh\left(\hat{\delta}\right) - \cosh\left(\hat{\delta}\left(1-\hat{y}\right)\right)}{\hat{\delta}\sinh\left(\hat{\delta}\right)}$

Critical Reynolds: $\mathrm{Re}_c^{\theta} = \dfrac{12\hat{\delta}\left(2+\hat{\delta}^2-2\hat{\delta}\coth\left(\hat{\delta}\right)\right)}{6\hat{\delta}\left(11+2\hat{\delta}^2\right)-\left(33+42\hat{\delta}^2\right)\coth\left(\hat{\delta}\right)+\hat{\delta}\left(33-2\hat{\delta}^2\right)\operatorname{csch}^2\left(\hat{\delta}\right)}$

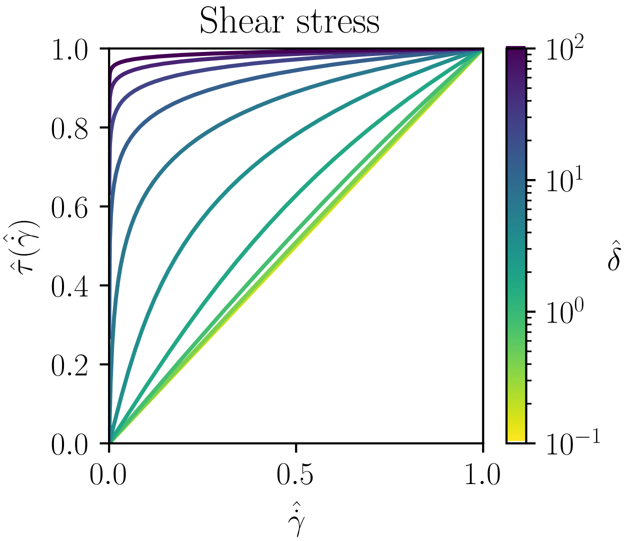

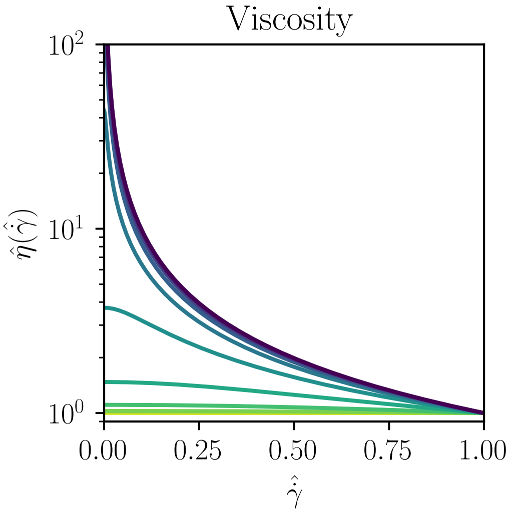

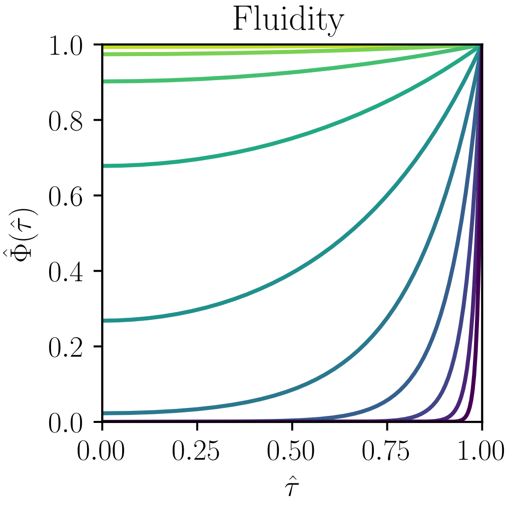

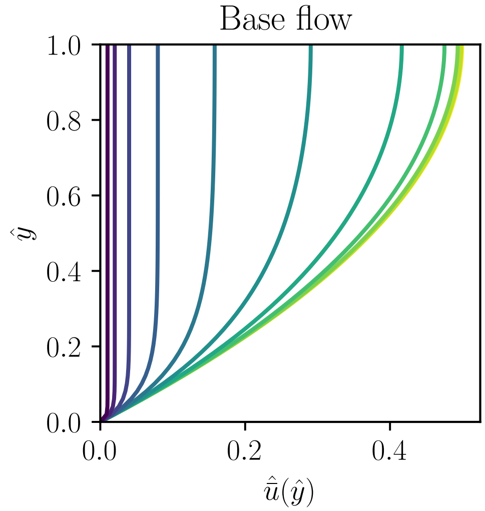

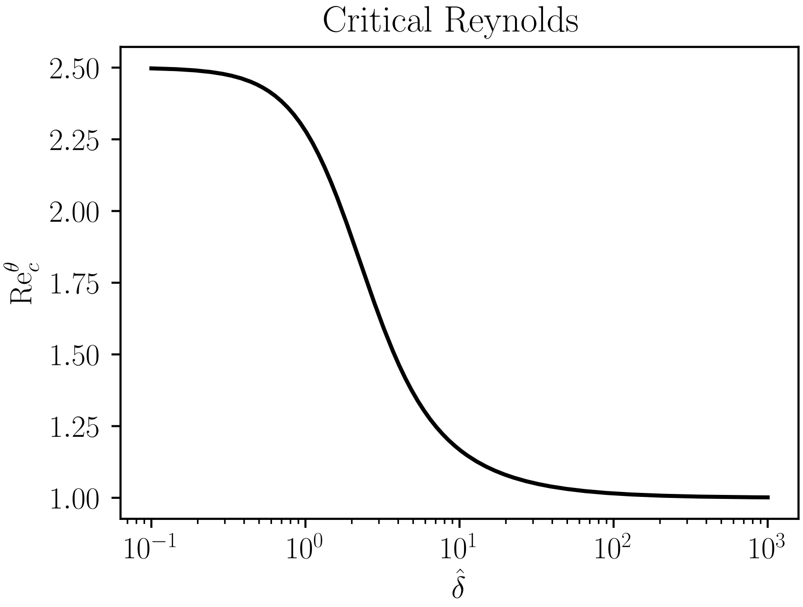

Supplement: S4 Fig — (PDF) [file pone.0310805.s006.pdf]
