# Supplementary material for: Unifying the roll waves
Source: PLoS One. 2024 Nov 19;19(11):e0310805. doi: 10.1371/journal.pone.0310805 (PMC11575793; doi:10.1371/journal.pone.0310805)

Wyart-Cates

Shear rate $\hat{\dot{\gamma}}(\hat{\tau}) = \left(\frac{\phi_J(\hat{\tau}) - \phi}{\phi_J(1) - \phi}\right)^2 \hat{\tau}$, with $\phi_J(\hat{\tau}) = \phi_0\left(1 - e^{\hat{\tau}^*/\hat{\tau}}\right) + \phi_1 e^{\hat{\tau}^*/\hat{\tau}}$, and $\phi_0 = 0.52$, $\phi_1 = 0.43$

Fluidity: $\hat{\Phi}(\hat{\tau}) = \left(\frac{\phi_J(\hat{\tau}) - \phi}{\phi_J(1) - \phi}\right)^2$

Viscosity: $\hat{\eta}(\hat{\tau}) = \left(\frac{\phi_J(1) - \phi}{\phi_J(\hat{\tau}) - \phi}\right)^2$

Base flow: $\hat{u}(\hat{y})$ numerically computed

Critical Reynolds: Re_c^θ numerically computed

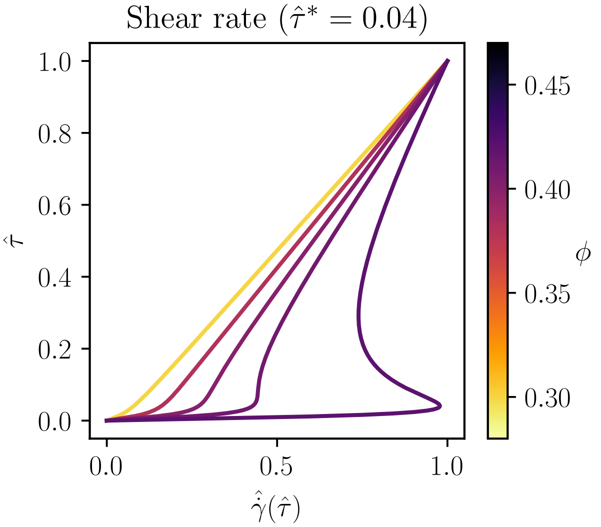

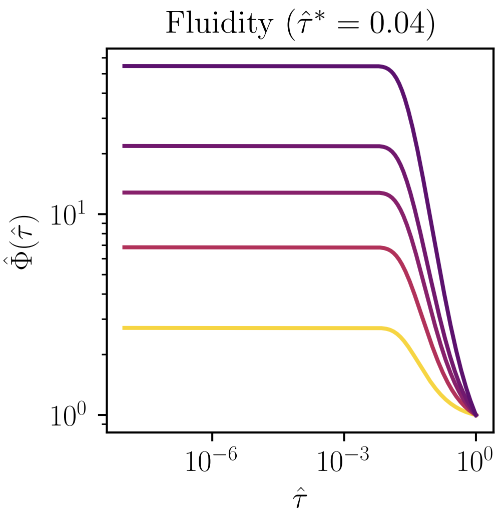

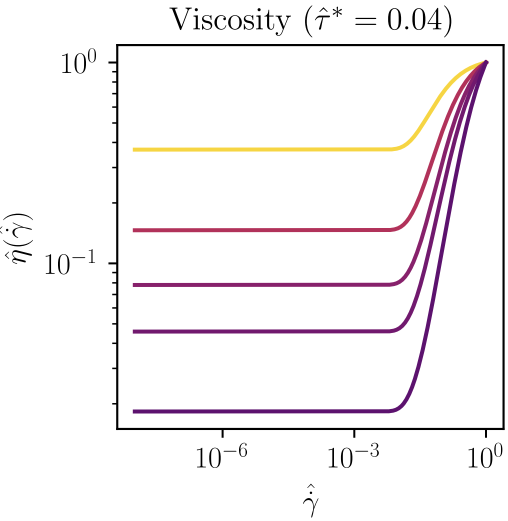

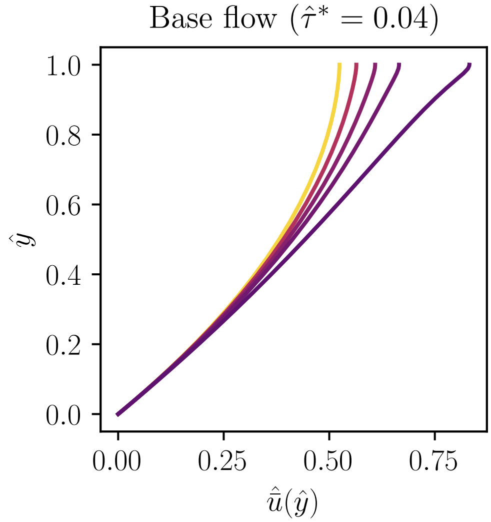

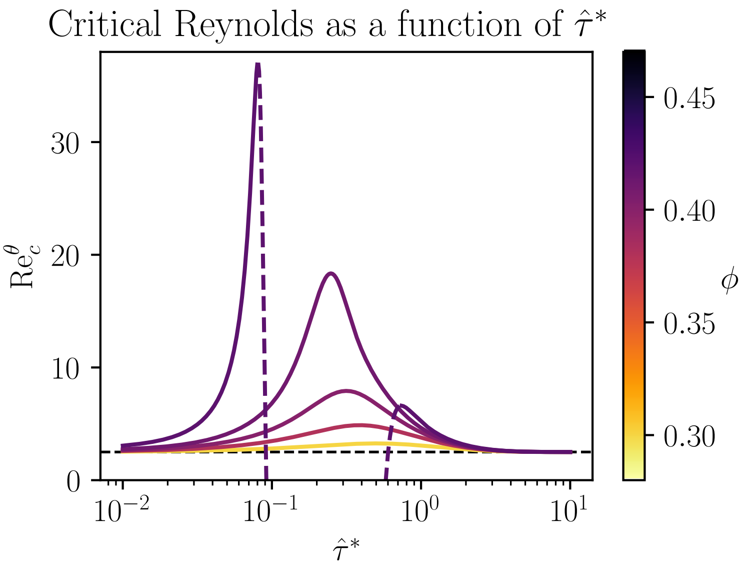

Supplement: S5 Fig — (PDF) [file pone.0310805.s007.pdf]
