# Supplementary material for: Unifying the roll waves
Source: PLoS One. 2024 Nov 19;19(11):e0310805. doi: 10.1371/journal.pone.0310805 (PMC11575793; doi:10.1371/journal.pone.0310805)

Herschel-Bulkley

Shear stress: $\begin{cases} \hat{\tau}\left(\hat{\dot{\gamma}}\right) = \mathrm{B} + (1-\mathrm{B})\,\hat{\dot{\gamma}}^{n} & \text{if } \hat{\tau} > \mathrm{B} \\ \hat{\dot{\gamma}} = 0 & \text{if } \hat{\tau} < \mathrm{B} \end{cases}$

Viscosity: $\hat{\eta}\left(\hat{\dot{\gamma}}\right) = \frac{\mathrm{B}}{\hat{\dot{\gamma}}} + (1-\mathrm{B})\,\hat{\dot{\gamma}}^{n-1} \quad \text{if } \hat{\tau} > \mathrm{B}$

Fluidity: $\hat{\Phi}\left(\hat{\tau}\right) = \frac{1}{\hat{\tau}}\left(\frac{\hat{\tau}-\mathrm{B}}{1-\mathrm{B}}\right)^{1/n}$

Base flow: $\hat{u}\left(\hat{y}\right) = \begin{cases} \frac{(1-\mathrm{B})^{m} - (1-\hat{y}-\mathrm{B})^{m}}{m\,(1-\mathrm{B})^{m-1}} & \text{if } \hat{y} \leq 1-\mathrm{B} \\ \frac{(1-\mathrm{B})^{m}}{m\,(1-\mathrm{B})^{m-1}} & \text{if } \hat{y} > 1-\mathrm{B} \end{cases}$ with $m = \frac{n+1}{n}$

Critical Reynolds: $\mathrm{Re}_c^{\theta} = \frac{(1+n+2n\mathrm{B}\,(n\mathrm{B}))\,(1+n)\,(2+n)\,(2+3n)}{2\,(2+n)\,(1+n)^{2} + n\,(2+n)\,(7+9n)\,\mathrm{B} + (11+19n+6n^{2})\,(2+n\mathrm{B})\,n^{2}\mathrm{B}^{2}}$

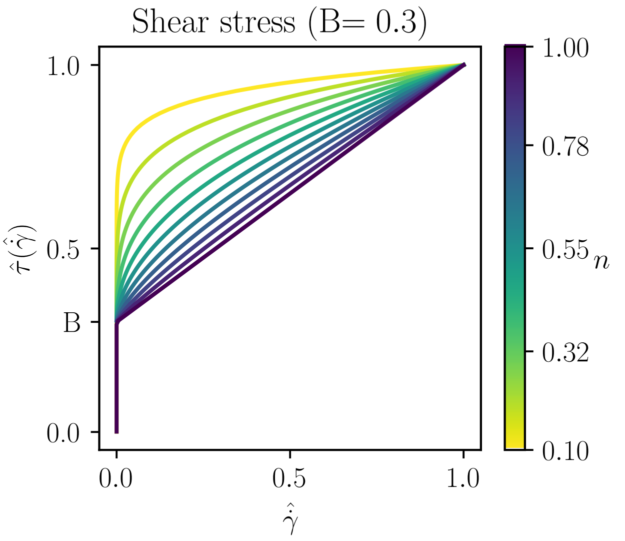

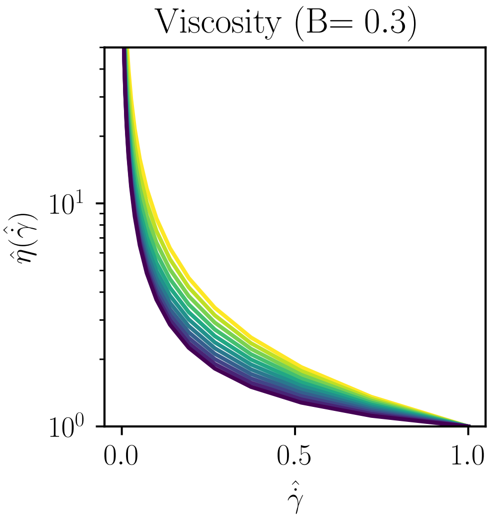

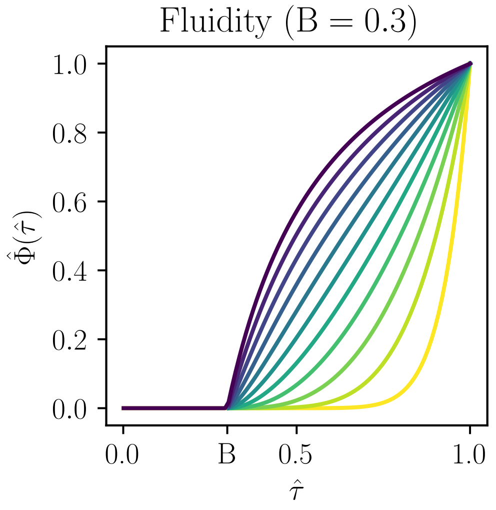

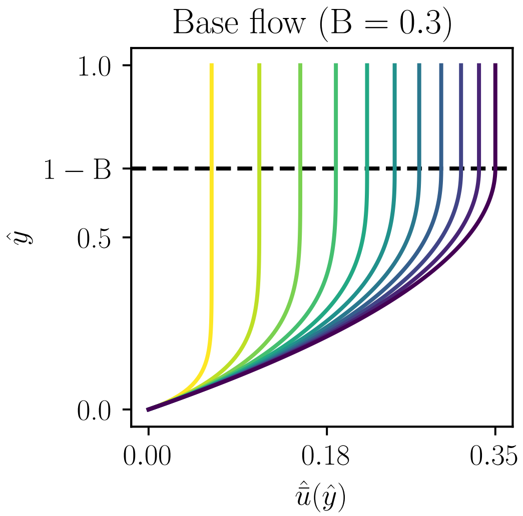

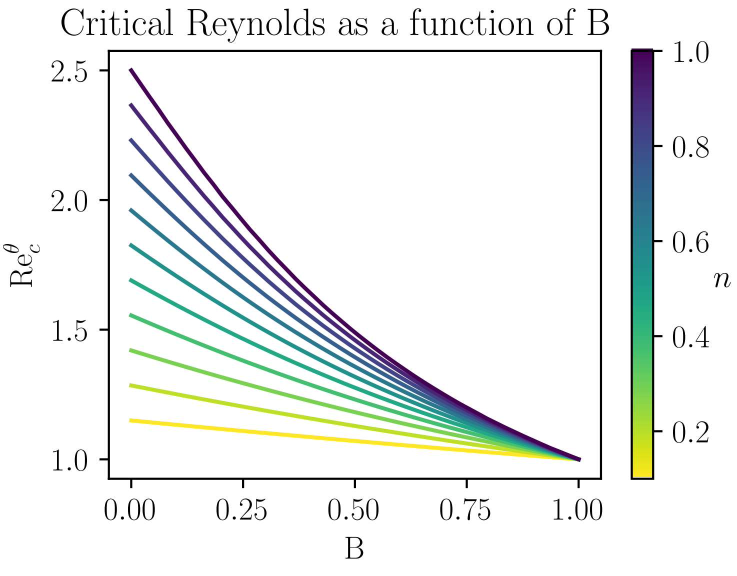

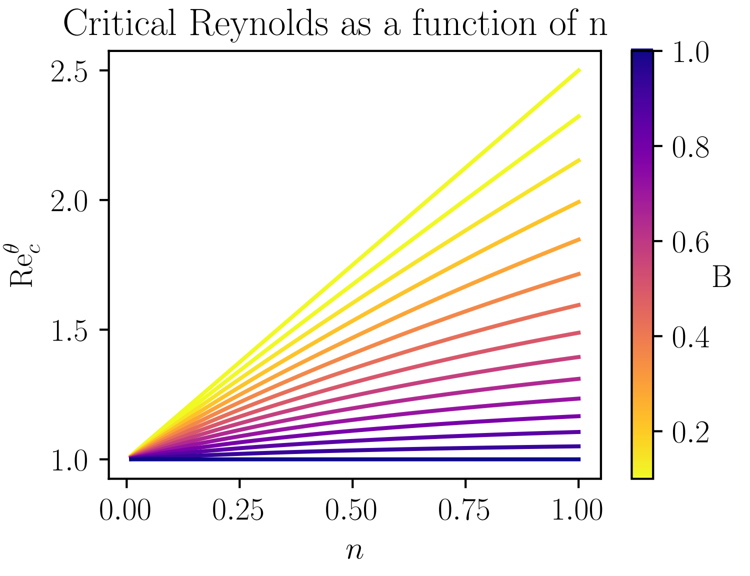

Supplement: S6 Fig — (PDF) [file pone.0310805.s008.pdf]
