# Supplementary material for: Unifying the roll waves
Source: PLoS One. 2024 Nov 19;19(11):e0310805. doi: 10.1371/journal.pone.0310805 (PMC11575793; doi:10.1371/journal.pone.0310805)

Williamson

Shear stress: $\hat{\tau}\left(\hat{\dot{\gamma}}\right) = \mathrm{B}\dfrac{\hat{\dot{\gamma}}}{\hat{\dot{\gamma}}+\epsilon} + \left(1-\dfrac{\mathrm{B}}{1+\epsilon}\right)\hat{\dot{\gamma}}^{n}$

Viscosity: $\hat{\eta}\left(\hat{\dot{\gamma}}\right) = \dfrac{\mathrm{B}}{\hat{\dot{\gamma}}+\epsilon} + \left(1-\dfrac{\mathrm{B}}{1+\epsilon}\right)\hat{\dot{\gamma}}^{n-1}$

Fluidity: $\hat{\Phi}\left(\hat{\tau}\right)$ numerically computed

Base flow: $\hat{u}\left(\hat{y}\right)$ numerically computed

Critical Reynolds: Re_c^{θ} numerically computed

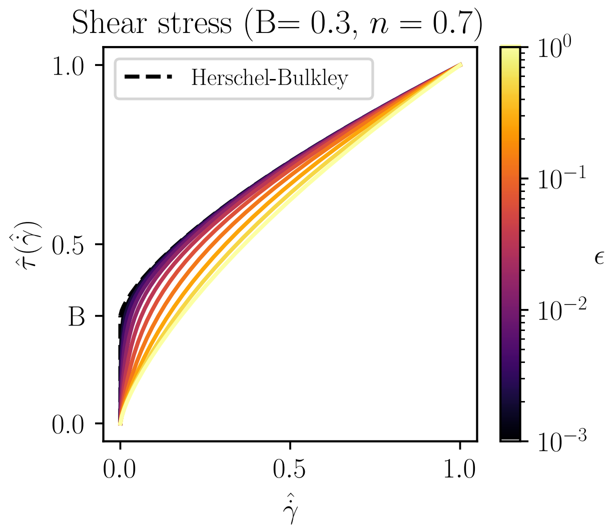

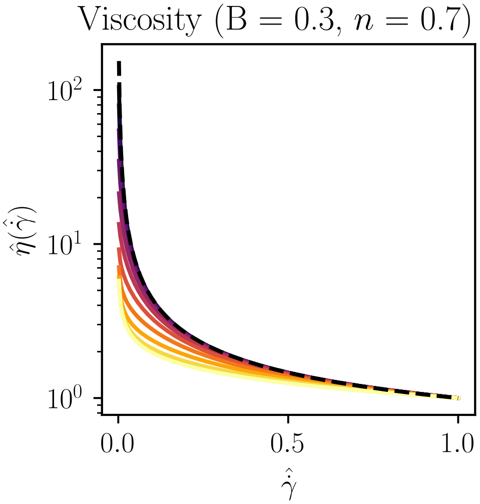

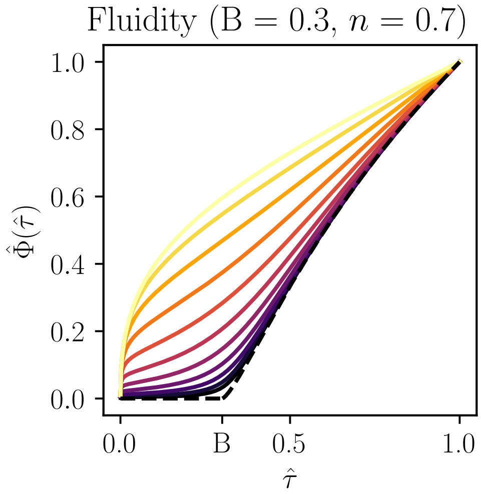

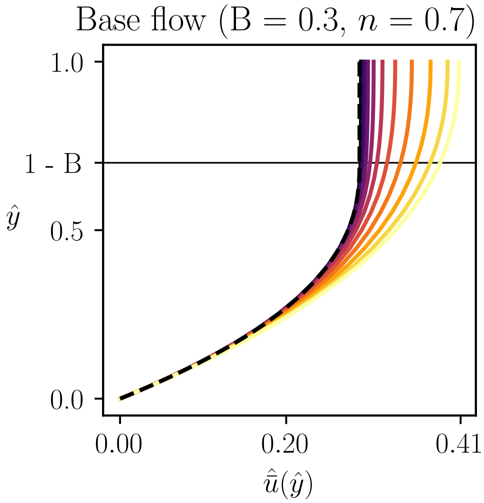

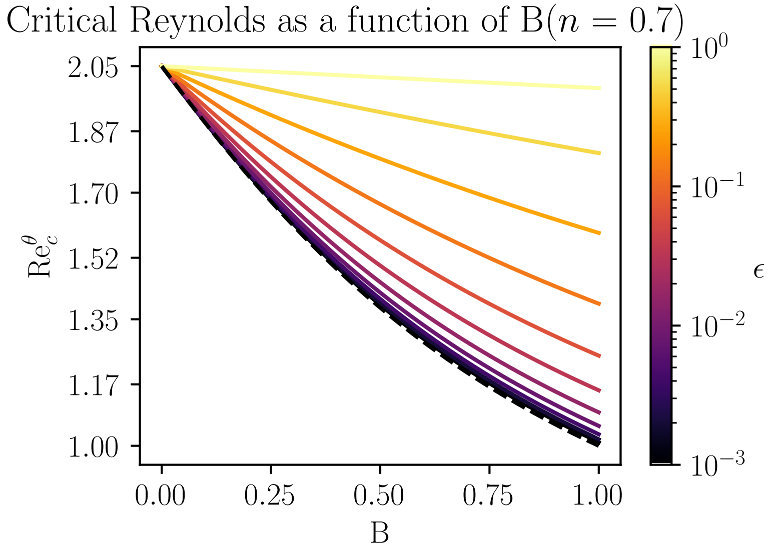

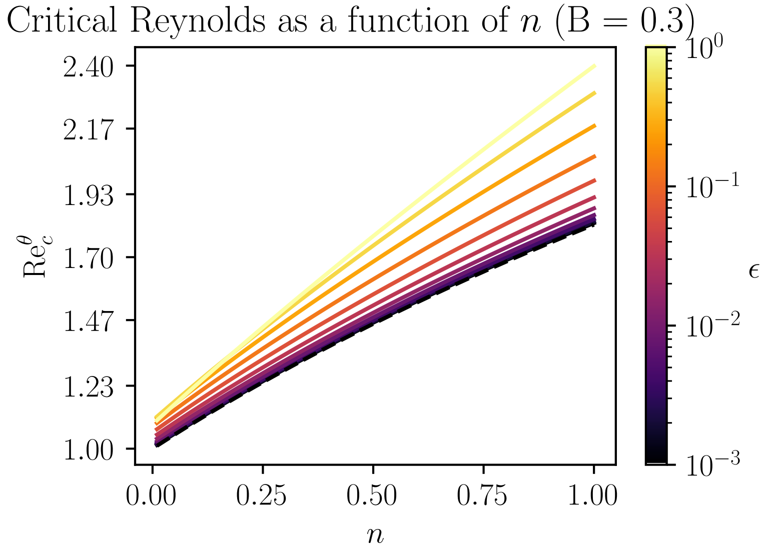

Supplement: S7 Fig — (PDF) [file pone.0310805.s009.pdf]
