# Supplementary material for: Unifying the roll waves
Source: PLoS One. 2024 Nov 19;19(11):e0310805. doi: 10.1371/journal.pone.0310805 (PMC11575793; doi:10.1371/journal.pone.0310805)

Papanastasiou

Shear stress: $\hat{\tau}\left(\hat{\dot{\gamma}}\right) = \mathrm{B}\left(1 - \exp\left(\frac{-\hat{\dot{\gamma}}}{\epsilon}\right)\right) + \left(1 - \left(1 - \exp\left(\frac{-1}{\epsilon}\right)\right)\right)\hat{\dot{\gamma}}^{n}$

Viscosity: $\hat{\tau}\left(\hat{\dot{\gamma}}\right) = \frac{\mathrm{B}}{\hat{\dot{\gamma}}}\left(1 - \exp\left(\frac{-\hat{\dot{\gamma}}}{\epsilon}\right)\right) + \left(1 - \left(1 - \exp\left(\frac{-1}{\epsilon}\right)\right)\right)\hat{\dot{\gamma}}^{n-1}$

Fluidity: $\hat{\Phi}\left(\hat{\tau}\right)$ numerically computed

Base flow: $\hat{u}\left(\hat{y}\right)$ numerically computed

Critical Reynolds: Re_c^{θ} numerically computed

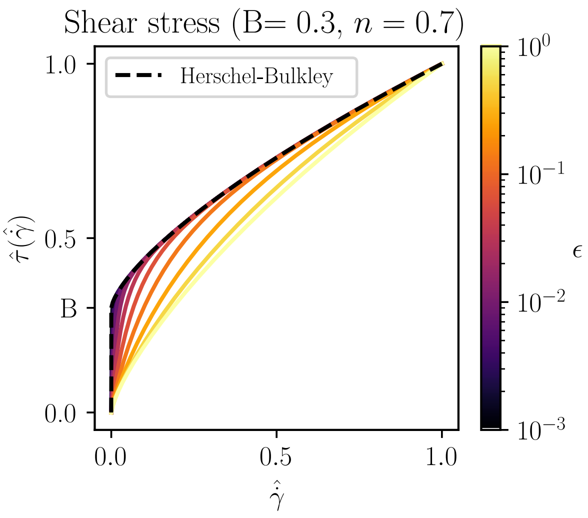

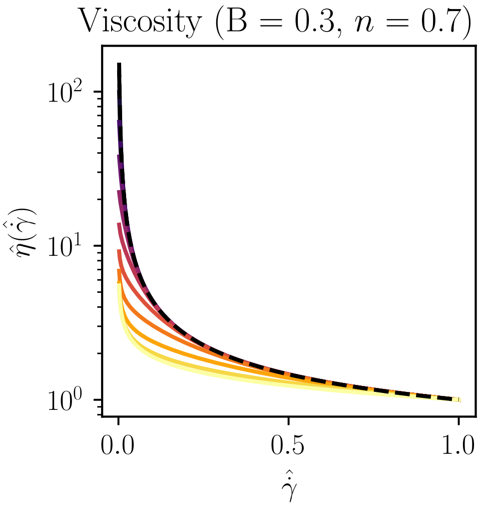

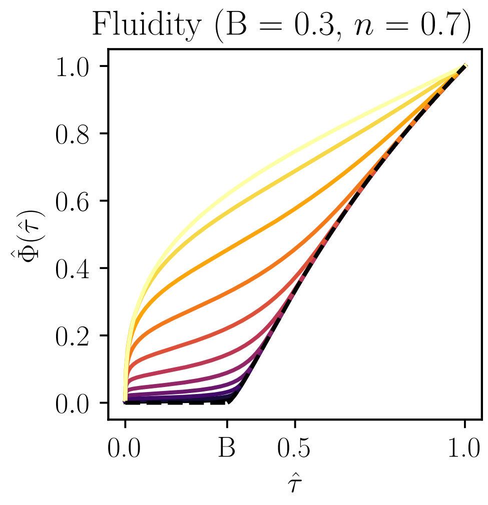

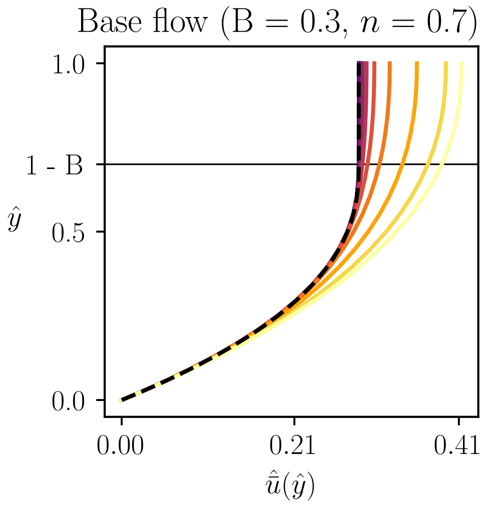

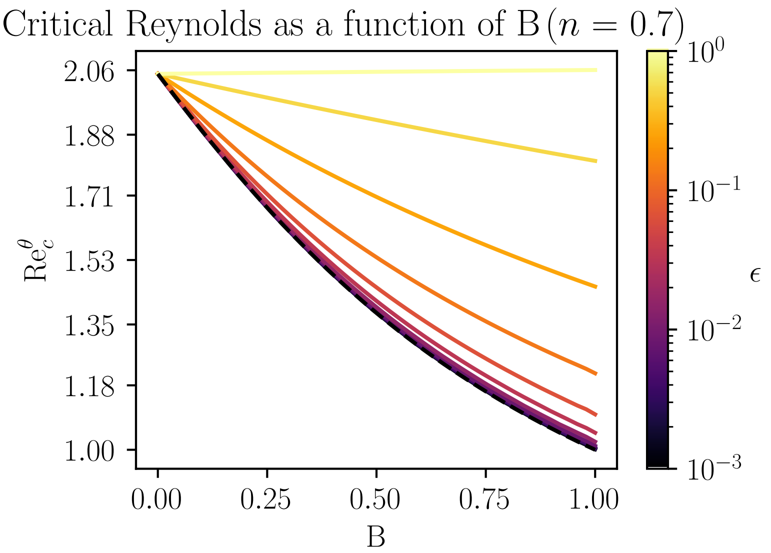

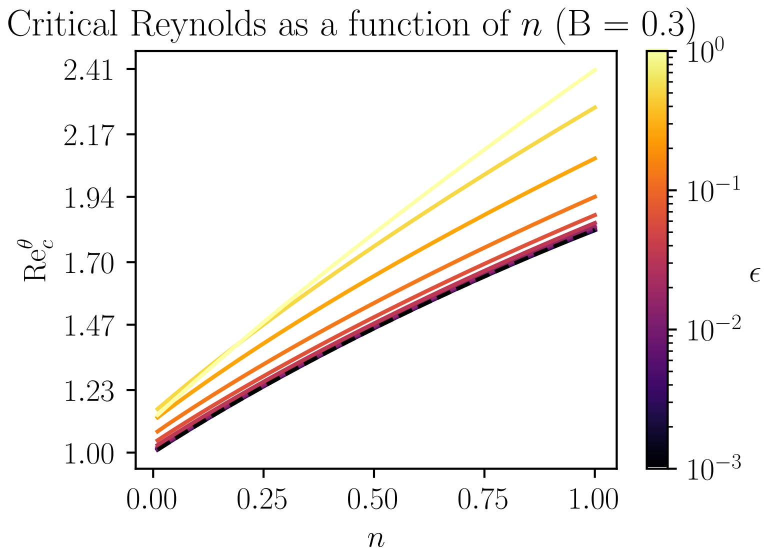

Supplement: S8 Fig — All these figures show the expressions of dimensionless shear stress τ^, viscosity η^, fluidity Φ^, base flow u¯^ and critical Reynolds number Recθ in their analytical form when it exists. Otherwise it is computed numerically. In all cases, these quantities are plotted as a function of the model rheological parameters. (PDF) [file pone.0310805.s010.pdf]
